# IL-33 facilitates proliferation of colorectal cancer dependent on COX2/PGE_2_

**DOI:** 10.1186/s13046-018-0839-7

**Published:** 2018-08-17

**Authors:** Yongkui Li, Jie Shi, Shanshan Qi, Jian Zhang, Dong Peng, Zhenzhen Chen, Guobin Wang, Zheng Wang, Lin Wang

**Affiliations:** 1 Research Center for Tissue Engineering and Regenerative Medicine, Union Hospital, Tongji Medical College, Huazhong University of Science and Technology, Wuhan, 430022 China; 2 Department of Gastrointestinal Surgery, Union Hospital, Tongji Medical College, Huazhong University of Science and Technology, Wuhan, 430022 China; 3 Department of Clinical Laboratory, Union Hospital, Tongji Medical College, Huazhong University of Science and Technology, Wuhan, 430022 China

**Keywords:** IL-33, Proliferation, Colorectal cancer, COX2, PGE_2_

## Abstract

**Background:**

Interleukin-33 (IL-33) participates in various types of diseases including cancers. Previous studies of this cytokine in cancers mainly focused on its regulation on immune responses by which IL-33 modulated cancer progression. The IL-33 triggered signals in cancer cells remain unclear.

**Methods:**

We analyzed IL-33 gene expression in human colorectal cancer (CRC) tissues and carried out gene enrichment analysis with TCGA Data Portal. We studied CRC proliferation in vivo by inoculating MC38 tumors in IL-33 transgenic mice. We investigated the cell proliferation in vitro with primary CRC cells isolated from fresh human CRC tissues, human CRC cell line HT-29 and mouse CRC cell line MC38. To evaluate the proliferation modulating effects of recombinant IL-33 incubation and other administrated factors, we measured tumor growth, colony formation, cell viability, and the expression of Ki67 and proliferating cell nuclear antigen (PCNA). We used several inhibitors, prostaglandin E2 (PGE_2_) neutralizing antibody, ST2 blocking antibody and specific shRNA expressing plasmid to study the pathway mediating IL-33-induced CRC proliferation. The IL-33 receptor ST2 in human CRC tissues was detected by immunohistochemistry staining and western blotting. The ST2-positive or negative subsets of primary CRC cells were acquired by flow cytometry sorting.

**Results:**

We found that IL-33 expression was correlated with the gene signature of cell proliferation in 394 human CRC samples. The MC38 tumors grew more rapidly and the tumor Ki67 and PCNA were expressed at higher levels in IL-33 transgenic mice than in wild-type mice. IL-33 promoted cell growth, colony formation and expression of Ki67 and PCNA in primary CRC cells as well as CRC cell lines. IL-33 activated cycloxygenase-2 (COX2) expression and increased PGE_2_ production, whereas the COX2 selective inhibitor and PGE_2_ neutralizing antibody abolished the proliferation promoting effect of IL-33. ST2 blockade, ST2-negative sorting, NF-κB specific inhibitor and NF-κB specific shRNA (shP65) abrogated the COX2 induction caused by IL-33.

**Conclusion:**

IL-33 facilitates proliferation of colorectal cancer dependent on COX2/PGE_2_. IL-33 functions via its receptor ST2 and upregulates COX2 expression through NF-κB signaling. Understanding the IL-33 signal transduction in CRC cells provides potential therapeutic targets for clinical treatment.

**Electronic supplementary material:**

The online version of this article (10.1186/s13046-018-0839-7) contains supplementary material, which is available to authorized users.

## Background

Interleukin-33 (IL-33), a pro-inflammatory cytokine, displays immunomodulatory functions by promoting inflammatory responses and driving Th2-type immune responses [1–3]. IL-33 mediates its biological effects mainly through specific receptor ST2, a member of Toll like receptor family [2, 4, 5]. IL-33 combined with ST2 stimulates numerous signal proteins by phosphorylation to mediate a series of physiological and pathological processes [1, 6]. IL-33 mediated pancreatic myofibroblast proliferation and migration by promoting IκBα and mitogen-activated protein kinase (MAPK) phosphorylation and inducing inflammatory mediators [7]. IL-33/ST2 axis promoted NF-κB-dependent IL-6 and IL-8 production in human fibroblasts [8]. IL-33 activated NF-κB signal in cardiomyocytes via upregrulating phenylephrine and angiotensin II to regulate cardiac fibrosis and hypertrophy [9]. IL-33/ST2 axis accelerated cytokine secretion from vascular endothelial cells to induce inflammatory reaction by activating extracellular signal-regulated kinase1/2 (ERK1/2) [10]. The increasing amount of evidence implies that IL-33-triggered signals may be involved in cancer progression. IL-33 is predominantly expressed in endothelial and epithelial cells [1, 11, 12]. Elevated levels of IL-33 protein were detectable in sera from non-small cell lung cancer (NSLC) patients, gastric cancer patients, hepatic carcinoma patients and metastatic pancreatic carcinoma patients [13–15]. Abnormally high IL-33 expression was also found in human colorectal cancer (CRC) tissues [4].

Previous studies showed IL-33 modulated tumor progression indirectly by regulating tumor stroma cells. IL-33/ST2 negatively regulated antitumor responses by promoting the function of regulatory T cells (Tregs) [16] or stimulating accumulation of myeloid-derived suppressor cells (MDSCs) [17, 18]. In oncogene-induced cholangio carcinoma, IL-33 stimulated cholangiocytes to produce the pro-tumorigenic cytokine IL-6 [19]. Recent studies revealed that IL-33 could directly regulate cancer cells [4, 20, 21]. Carcinoma-associated fibroblasts-derived IL-33 promoted cancer cell epithelial-to-mesenchymal transdifferentiation (EMT) to regulate head and neck squamous cell carcinoma invasion and migration [22]. The function of IL-33/ST2 axis in cancer cells is poorly understood.

IL-33-associated inflammation has profound influence on tumorigenesis of CRC [23, 24]. IL-33/ST2 signal impaired permeability of epithelial barrier and triggered immune cells to produce IL-6 during CRC progression [25, 26]. Stroma-derived IL-33 drove CRC neoplastic transformation from adenoma to carcinoma by promoting angiogenesis [27]. IL-33 induced CRC carcinogenesis and liver metastasis by remodeling tumor microenvironment and activating angiogenesis [28]. In this study, we found that IL-33 was positively correlated with proliferation of CRC both in human data and in transgenic mice. We further investigated the direct proliferation promoting role of IL-33 with primary CRC cells and CRC cell lines.

## Methods

### TCGA data and statistics analysis

The global gene expression data of 394 colorectal cancer samples were acquired from the Cancer Genome Atlas (TCGA) database (https://gdc.cancer.gov/). The clinical information of CRC patients was listed in Additional file 1: Table S1. Gene expression levels of IL-33, ST2 and COX2 included in the data were subjected to Kolmogorov-Smirnov (K-S) test of normality. The gene set enrichment analysis (GSEA) was performed using the program GSEA v2.2.0. Gene sets for GSEA were obtained from the Molecular Signature Database (MSigDb) (http://www.broadinstitute.org/gsea/msigdb/index.jsp.). The Log2-rank test was used to make gene enrichment statistical comparisons. *P*-value (*P* < 0.05) was regarded statistically significant. Pearson’s correlation test was performed using SPSS software with COX2 and ST2 expression levels extracted from the downloaded data. *P*-value (*P* < 0.05) was regarded statistically significant. For experimental data, statistical analysis was performed using the GraphPad Prism 5 software. Student's t-test was used for comparison of paired groups. Multiple group comparisons were performed using analysis of variance, ANOVA.

### Reagents

PGE_2_ and human recombinant IL-33 were purchased from ProteinTech. The mouse recombinant IL-33 was purchased from Pepro Tech. The following antibodies were used: ST2 antibody (R&D systems), PGE_2_ antibody (Cayman), COX2 antibody (Abclonal), and control IgG (Santa Cruz). The following chemical reagents were used: SB203580 (Cayman, 10 μg/mL), PD98059 (Cayman, 20 μg/mL), SP600125 (Cayman, 10 μg/mL), BIX01294 (MCE, 2 μM), 5-Aza (Sigma, 10 μM), SC-560 (Cayman, 0.1 μg/mL), Celecoxib (Sigma, 20 μg/mL) and BAY11–7082 (Cayman, 10 μM).

### Cell lines and animals

Primary CRC cell lines were isolated as described previously [4, 29] from fresh human CRC tissues of three patients. Human CRC cell line HT29 was bought from American Type Culture Collection (ATCC). Murine CRC cell line MC38 was provided by Dr. Weiping Zou (Michigan, USA) and was tested in 2011 [30]. These cells were all cultured in RPMI1640 medium with 10% fetal bovine serum, 100 IU/mL penicillin and 100 μg/mL streptomycin at 37 °C in a cell incubator with a 5% (*v*/v) CO2 humidified atmosphere. C57/BL6 wild type mice were bought from Beijing HFK Bioscience Co. Ltd. C57/BL6 IL-33 transgenic mice were from Dr. Zhanguo Li (Beijing, China) and Dr. Lianfeng Zhang (Beijing, China) [4]. All mice were housed in specific pathogen free (SPF) animal room of Tongji Medical College.

### Animal models

Six-week-old IL-33 transgenic mice and wild-type male C57/BL6 mice were used in tumor growth experiments. Each mouse was inoculated with 1 × 10^6^ MC38 cells subcutaneously on the back. Once visible tumors generated, tumor sizes were measured every 2 days. Tumor volumes were calculated by the formula V = 1/2 × length×width^2^ (mm^3^). For comparing the tumor growth rates, seven mice were set in each group. Another same set of experiment was performed for harvesting tumor tissues.

### Immunohistochemistry

Immunohistochemistry staining was performed as described previously [31]. The tumors removed from the wild-type or IL-33 transgenic mice at Day 22 post tumor inoculation were fixed with 4% formaldehyde and embedded with paraffin. The sections were labeled with anti-Ki67 antibody (Arigo, 1:200) and anti-PCNA antibody (Boster, 1:200). Quantification of Ki67 and PCNA expression was independently performed by two pathologists. The positively staining cells were quantified by ImageJ software. Twenty CRC tissues and adjacent normal tissues were obtained from surgery in Union hospital (Wuhan) with the permission of each patient. The ST2 staining was performed with anti-ST2 antibody (R&D, 1:200).

### Measurement of cell viability

Primary CRC cells were seeded in 96-well plates (6000 cells per well) and incubated with RPMI1640 medium with IL-33 (0, 50, 100, 200 ng/mL) or PGE_2_ (50 ng/mL). Cell viability was measured with the Cell Counting Kit-8 (Biosharp) at 24^th^, 48^th^ or 72^nd^ h. The curves of cell viability were plotted by the absorbance of each time point.

### Real-time quantitative PCR

Primary CRC cells, HT29 cells or MC38 cells were seeded in 12-well plates (2 × 10^5^ cells per well) and incubated with the following reagents: human/mouse recombinant IL-33 proteins (0, 50 or 100 ng/mL), celecoxib (20 μg/mL), ST2 antibody (2 μg/mL) or BAY11–7082 (10 μM) for 24 h. Three parallel wells were set for each treatment. Total RNA was isolated with TRIzol reagent (Invitrogen) and was reversely transcribed into complementary DNA with RNA reverse transcriptase (Vazyme). Real-time PCR was performed on ABI StepOne Plus Detector System (Applied Biosystem). Relative mRNA expression of human genes was normalized to GAPDH, and for mouse genes the mRNA levels were normalized to mouse Hprt gene. Each experiment was repeated three times and representative results were shown. The primers used are listed in Additional file 1: Table S2.

### Flat colony formation

CRC cells were seeded in 12-well plates at a density of 500 viable cells per well. Then the cells were incubated in RPMI1640 medium with recombinant IL-33 protein (added at Day 1, 3 and 5), celecoxib, the ST2 antibody or the BAY11–7082. Colonies were photographed and counted at Day 10 or 15 to allow all wells undergoing different treatments to generate visible colonies. Three parallel wells were set for each treatment. Each experiment was repeated three times and representative results were shown.

### Western blotting

Primary CRC cells, HT29 cells or MC38 cells were seeded in 6-well plates (5 × 10^5^ cells per well). The CRC cells receiving different treatments were scraped and collected by low speed centrifugation and lysed using Cell Lysis Buffer. The Western blotting was performed as previously described [32]. Blots were performed with a COX2 antibody (Abcolonal), ST2 antibody (R&D systems), NF-κB P65 antibody (CUSABIO) and β-actin antibody (Proteintech). Specific bands were detected using ECL detection reagents (Millipore, USA). Each experiment was repeated three times and representative results were shown.

### ELISA for quantification of PGE_2_

Primary CRC cells seeded in 6-well plates (5 × 10^5^ cells per well) were incubated in RPMI1640 medium or RPMI1640 medium containing rhIL-33 protein (100 ng/mL) for 24 h. The culture supernatants were collected. The concentration of PGE_2_ was measured by the Parameter^TM^ PGE_2_ assay kit (R&D) according to the manufacturer’s instructions.

### Flow cytometry analysis and sorting

Primary CRC cells (5 × 10^6^) were collected from culture plates by low speed centrifugation and made into signal-cell suspensions. Primary CRC cells were stained with PE-conjugated specific antibody to ST2 (bs-2382R, Bioss, China) and PE-conjugated isotype IgG (bs-0295P-PE, Bioss, China). The sample was diluted into a concentration of 2 × 10^6^ cells/mL for sorting. The sorting was performed by a high speed flow sorter (FACSAria II, BD). The sorting system was fluxed using ethanol 70% for 10 min to reduce pollution. Sheath fluid was using autoclaved and filtered (0.22 μm) phosphate saline buffer (1 × PBS). Sorting rate typically was 3000 events/s, and cells were acquired at a rate of 300–500 cells/s. By comparing to the negative control, the PE positively stained subset was gated for sorting as the ST2-positive subset of primary CRC cells. The rest cells were collected and used as ST2-negative primary CRC cells. Cells were recovered into a 15 mL Eppendorf tube with washing buffer. Before being used for experimental assay, the sorted ST2-positive and ST2-negative cells were subjected to flow cytometry analysis with the same program and testing with the same gating condition used for sorting. Purity of sorted cell subsets was > 90% as verified by flow cytometry. Flow cytometry data was analyzed by Flow Jo 7.6.1 software.

## Results

### IL-33 promotes the proliferation of CRC cells

**Fig. 1:**
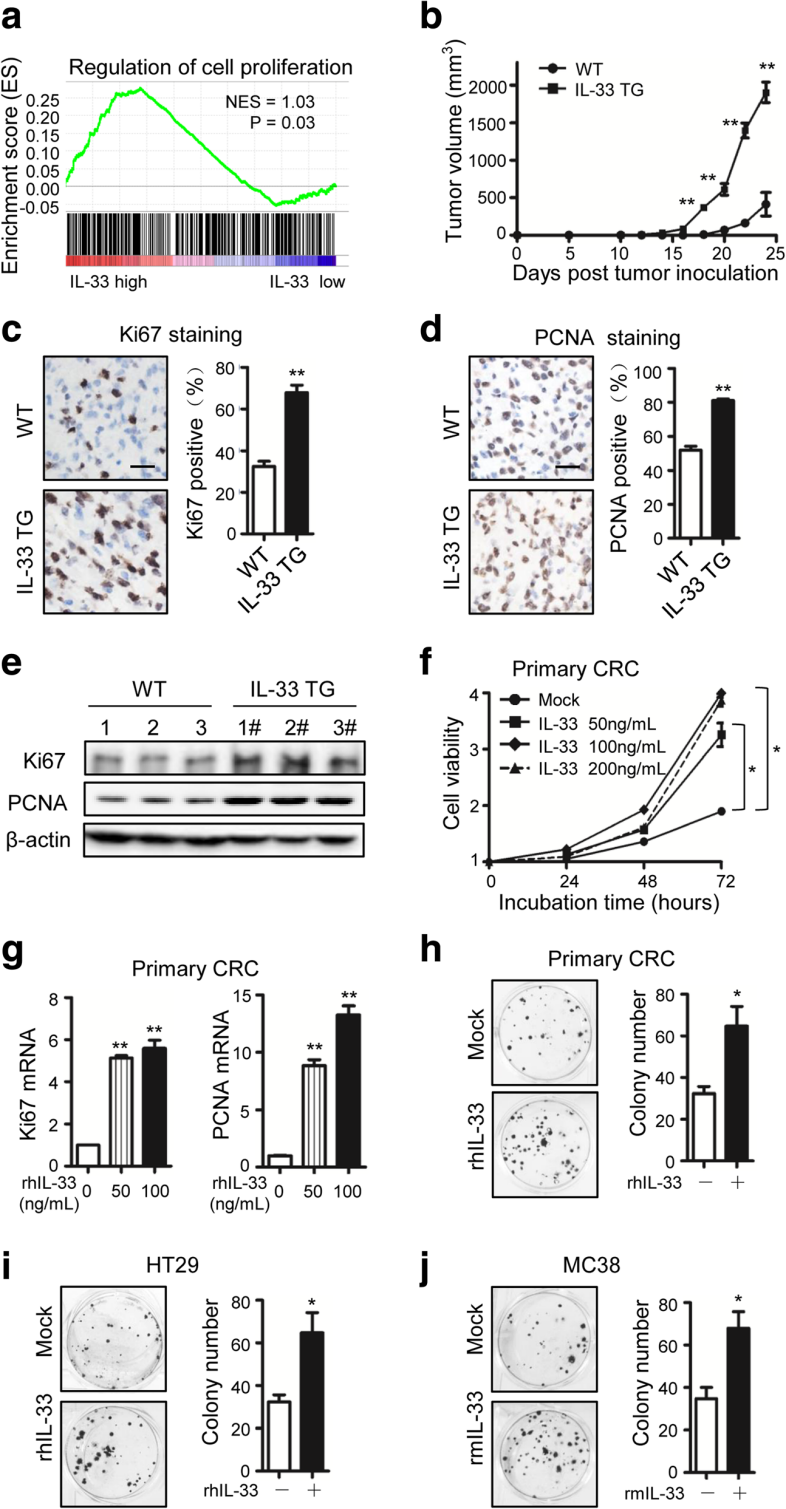
IL-33 promotes CRC proliferation both in vivo and in vitro. **a** Correlation between IL-33 transcripts and the genes involved in the regulation of cell proliferation in CRC. Gene set enrichment analysis was performed using CRC TCGA database. NES = 1.03, *P* = 0.03. **b** Growth curves of MC38 tumors inoculated in IL-33 transgenic mice (IL-33 TG) or wild-type mice (WT). *n* = 7. **c**, **d** Immunohistochemical staining of Ki67 (**c**) and PCNA (**d**) in the MC38 tumors recovered from wild-type and IL-33 transgenic mice at Day 22 post inoculation. The representative images and the statistical proportions of positive cells are shown. Scale bar, 50 μm. *n* = 7. Data expressed as mean ± SEM. **, *P* < 0.01. **e** Western blot of Ki67 and PCNA in the MC38 tumors recovered from wild-type and IL-33 transgenic mice. *n* = 3. **f** Cell viabilities of human primary CRC cells incubated with 0, 50, 100 or 200 ng/mL of rhIL-33 in medium at 24^th^, 48^th^ and 72^nd^ h. Six parallel wells were set for each treatment. The experiment was performed three times. Data expressed as mean ± SEM. * *P* < 0.05. **g** Ki67 and PCNA mRNA levels in primary CRC cells incubated with rhIL-33 (0, 50 or 100 ng/mL) for 24 h. Each experiment was performed three times. Three parallel wells were set for each treatment. Data expressed as mean ± SEM. ** *P* < 0.01. **h**, **i**, **j** The flat colony formation with 500 primary CRC cells (**h**) and 500 HT29 cells (**i**) incubated with rhIL-33 (100 ng/mL) and the flat colony formation with 500 MC38 cells (**j**) incubated with rmIL-33 (100 ng/mL). The number of colony was counted at Day 10. Each experiment was performed three times. Three parallel wells were set for each treatment. The representative images of colonies and the statistical data are shown. Data expressed as mean ± SEM. * *P* < 0.05

To investigate the signaling of IL-33 in CRC, we analyzed the gene expression data from TCGA Data Portal that consisted of 394 CRC samples. Enrichment analysis revealed that the gene signature of cell proliferation was significantly correlated with IL-33 expression (Fig. 1a; Additional file 1: Table S3). This indicates that IL-33 might regulate the proliferation of CRC cells. Thus, we performed experiments to test this notion. By inoculating MC38 CRC cells in animals, we found the tumor growth in IL-33 transgenic mice was more rapidly than that in wild-type mice (Fig. 1b). The immunohistochemical staining showed that the cell proliferation marker Ki67 and the proliferating cell nuclear antigen (PCNA) were expressed significantly higher in the tumors generated in IL-33 transgenic mice than in the tumors from wild-type mice (Fig. 1c, d). The increased Ki67 and PCNA expression in the tumors from IL-33 transgenic mice were verified by Western blotting (Fig. 1e). To determine whether IL-33 facilitated the proliferation of CRC cells directly or via regulating other factors in vivo, we incubated primary CRC cells isolated from human cancer tissues with recombinant IL-33 protein. We found that IL-33 increased cell viability of primary CRC cells and upregulated the expression of Ki67 and PCNA in a dose dependent manner (Fig. 1f, g). To confirm the direct effects of IL-33 on the proliferation of CRC cells, we performed colony formation assay with human CRC cell line HT-29, mouse CRC cell line MC38 as well as the primary human CRC cells. IL-33 significantly facilitated the colony formation of all the three types of cells (Fig. 1h, i, j). Therefore, we concluded that IL-33 accelerated proliferation of CRC.

### IL-33 facilitates CRC proliferation dependent on COX2/PGE_2_

**Fig. 2:**
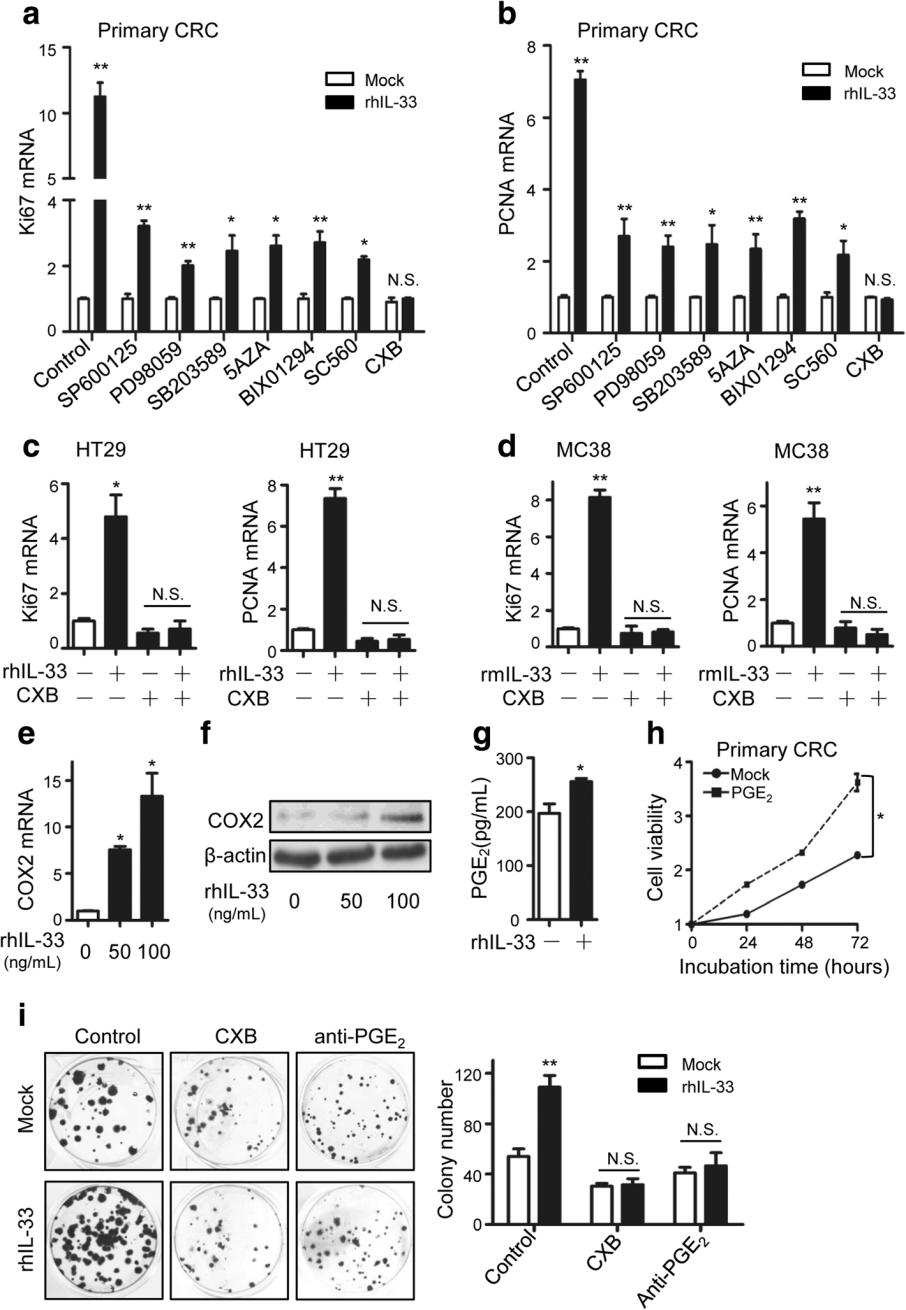
COX2/PGE_2_ mediates the proliferation promoting function of IL-33. **a**, **b** The relative mRNA levels of Ki67 (**a**) and PCNA (**b**) in primary CRC cells responding to rhIL-33 (100 ng/mL) incubation and/ or indicated inhibitors (SB203580, 10 μg/mL; PD98059, 20 μg/mL; SP600125, 10 μg/mL; BIX01294, 2 μM; 5Aza, 10 μM; SC560, 0.1 μg/mL; celecoxib, 20 μg/mL) for 24 h. **c** The relative mRNA levels of Ki67 and PCNA in HT-29 cells incubated with rhIL-33 (100 ng/mL) or/ and celecoxib (CXB) (20 μg/mL) in medium for 24 h. **d** The relative mRNA levels of Ki67 and PCNA in MC38 cells incubated with rmIL-33 (100 ng/mL) or/ and celecoxib (CXB) (20 μg/mL) in medium for 24 h. **e**, **f** The mRNA (**e**) and protein (**f**) expression of COX2 in primary CRC cells incubated with 0, 50 or 100 ng/mL of rhIL-33 in medium for 24 h. **g** PGE_2_ concentrations in the supernatants of primary CRC cells incubated with rhIL-33-contained RPMI medium or blank RPMI medium for 48 h. **h** Cell viabilities of primary CRC cells incubated with or without PGE_2_ (50 ng/mL) in medium. **i** The flat colony formation of primary CRC cells incubated for 15 days in medium containing different factors as indicated (IL-33, 100 ng/mL; celecoxib, 20 μg/mL; anti-PGE_2_, 2 μg/mL). The representative images of colonies and the statistical data are shown. Three parallel wells were set for each treatment. Each experiment was performed three times. Data expressed as mean ± SEM. * *P* < 0.05. ** *P* < 0.01

We next sought to investigate the mechanism how IL-33 facilitated CRC proliferation. We screened tumor proliferation associated signals: DNA and histone methylation and prostaglandin E2 (PGE_2_) synthesis using inhibitors. The IL-33-induced Ki67 and PCNA were detected when the primary CRC cells were treated with the P38 inhibitor SB203580, the MAPK/ERK kinase (MEK) inhibitor PD98059, the c-Jun N-terminal kinase (JNK) inhibitor SP600125, the histone methyltransferase inhibitor BIX01294, the DNA methyltransferase inhibitor 5-Aza, COX1 selective inhibitor SC-560, and the COX2 selective inhibitor celecoxib. We found that in celecoxib treated primary CRC cells IL-33 did not elevate Ki67 or PCNA (Fig. 2a, b). In CRC cell lines HT-29 and MC38, celecoxib also effectively abrogated the IL-33-induced elevation of Ki67 and PCNA (Fig. 2c, d). COX2 functions as a key enzyme in the synthesis of PGE_2_ that potently accelerates tumor proliferation [33–35]. These indicate that COX2/PGE_2_ might mediate the proliferation promoting function of IL-33. In accordance with this notion, IL-33 incubation increased COX2 mRNA and protein levels in the primary CRC cells in a dose dependent manner (Fig. 2e, f). CRC cells incubated with IL-33 produced significantly higher level of PGE_2_ (Fig. 2g). The artificially synthesized PGE_2_ increased the cell viability of the primary CRC cells (Fig. 2h), verifying its function in promoting tumor proliferation characterized previously. To confirm the autocrine of PGE_2_ mediated the IL-33-induced acceleration of proliferation, we performed colony formation with CRC cells incubated with a PGE_2_ neutralizing antibody as well as the inhibitor celecoxib. Both PGE_2_ neutralizing antibody and celecoxib blocked the increase of colony numbers induced by IL-33 (Fig. 2i). Together, IL-33 facilitated CRC proliferation via increasing PGE_2_ production.

### The receptor ST2 mediates the proliferation promoting function of IL-33

**Fig. 3:**
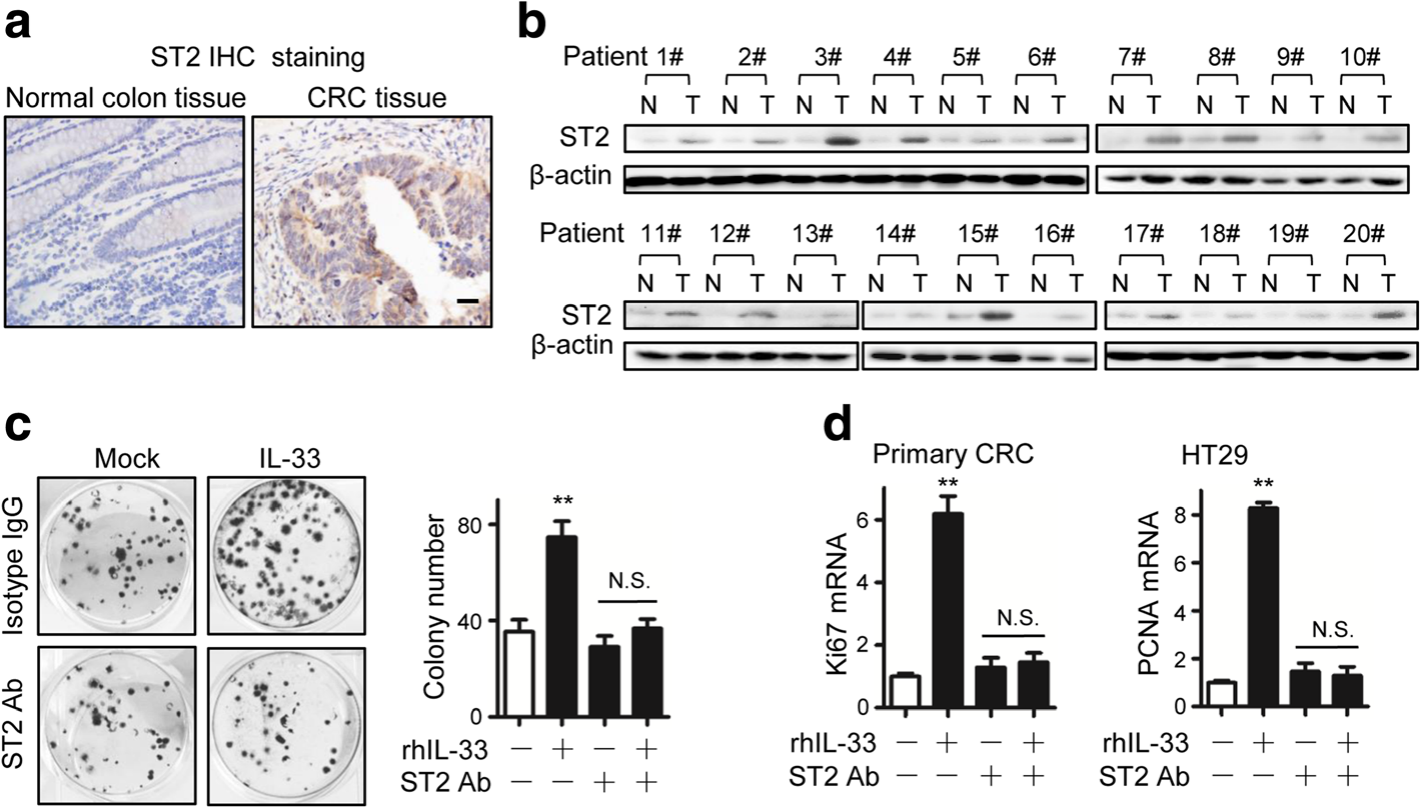
IL-33 facilitated CRC proliferation by signaling its receptor ST2. **a** Immunohistochemical staining of ST2 in the CRC tissues and the adjacent normal colon tissues (20 pairs). The representative images are shown. Scale bar, 20 μm. **b** ST2 expression levels in the paired CRC tissues (T) and the adjacent normal colon tissues (N) analyzed by Western blotting. **c** The flat colony formation of primary CRC cells incubated for 15 days in RPMI medium or RPMI medium containing rhIL-33 (100 ng/mL) or/ and ST2 antibody (2 μg/mL). Three parallel wells were set for each treatment. Each experiment was performed three times. The representative images of colonies and the statistical data are shown. Data expressed as mean ± SEM. ** *P* < 0.01. **d** Ki67 and PCNA expression in primary CRC cells responding to the incubation with rhIL-33 (100 ng/mL) or/ and ST2 antibody (2 μg/mL) for 24 h. Three parallel wells were set for each treatment. Each experiment was performed three times. Data expressed as mean ± SEM. ** *P* < 0.01

We previously reported that cultured CRC cells expressed functional IL-33-recceptor ST2 [4]. Here we further detected the receptor in human CRC. By immunohistochemical staining, we found positive ST2 expression in most of  CRC samples (19/20), whereas ST2-positive staining was rarely observed in the adjacent normal tissues (Fig. 3a). The ST2 expression in CRC tissues was also verified by Western blotting (Fig. 3b). Although ST2 has been identified as IL-33 receptor, IL-33 can function in an ST2-independent fashion [36]. We checked whether IL-33 promoted CRC proliferation through its receptor by using an ST2 blockade antibody. ST2 blockade abolished the increase of colony numbers caused by IL-33 incubation (Fig. 3c). The antibody treatment also suppressed the IL-33-elevated Ki67 and PCNA levels (Fig. 3d). Thus, we demonstrated that IL-33 facilitates CRC proliferation by signaling through its receptor ST2.

### IL-33/ST2 axis induces COX2 expression by activating NF-κB signaling

**Fig. 4:**
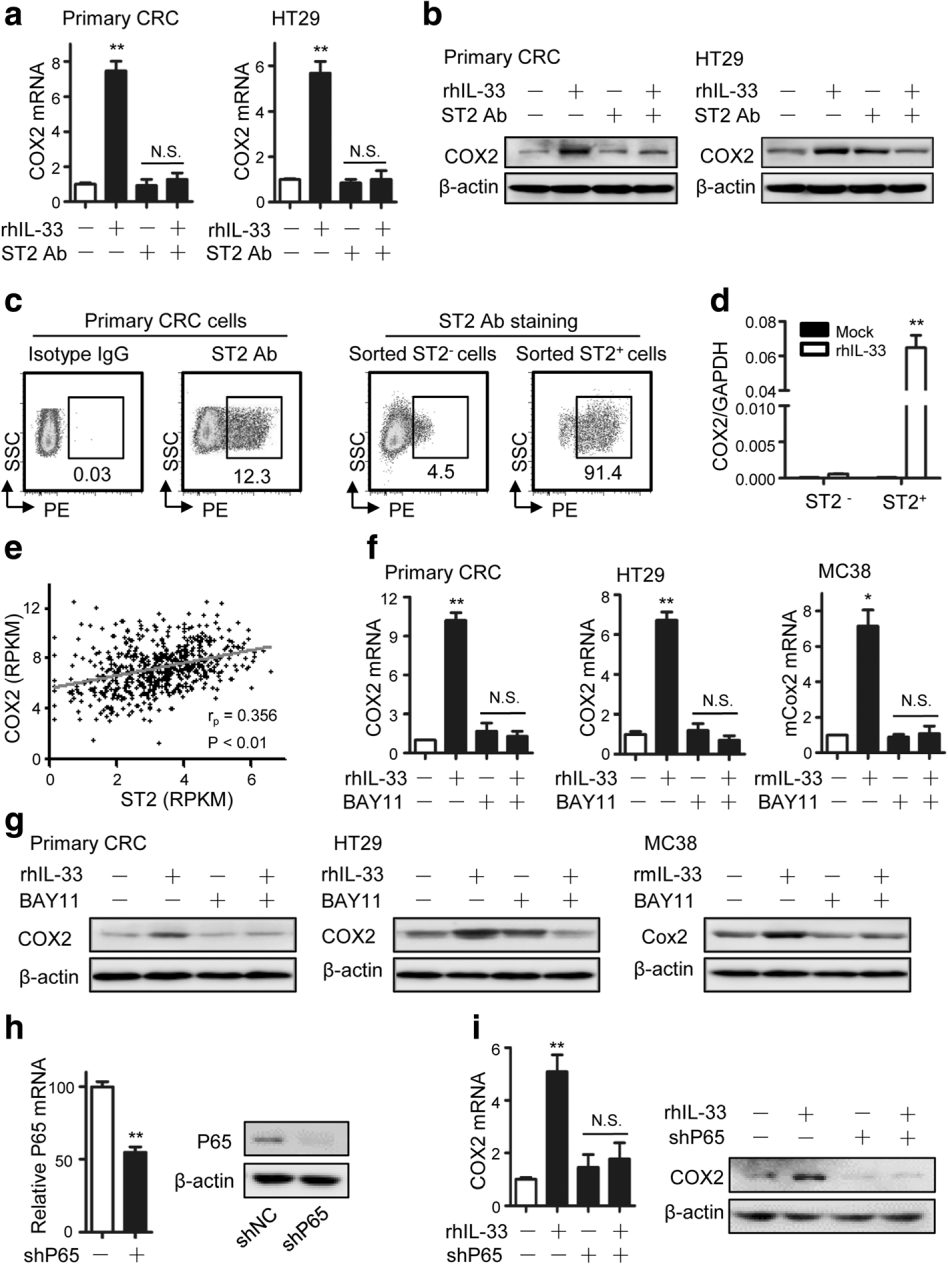
IL-33/ST2 upregulates COX2 expression through NF-κB signaling. **a**,**b** The COX2 mRNA (**a**) and protein (**b**) expression in primary CRC cells or HT29 cells responding to the incubation with rhIL33 (100 ng/mL) or/ and ST2 antibody (2 μg/mL) for 24 h. Each experiment was performed three times. Data expressed as mean ± SEM. ** *P* < 0.01. **c** ST2 expression distribution in primary CRC cells, sorted ST2-negative and sorted ST2-positive primary CRC cells. The proportion of ST2 positive subset is shown. **d** Relative COX2 mRNA levels in ST2-negative or ST2-positive primary CRC cells responding to IL-33 (100 ng/mL) incubation for 24 h in 24-well plates (1 × 10^5^ cells per well). Three parallel wells were set for each treatment. Data expressed as mean ± SEM. ** *P* < 0.01. **e** The correlation between COX2 and ST2 transcripts in 394 CRC samples recorded in TCGA database. These two sets of data both have a normal distribution. Pearson *r* = 0.356, *P* < 0.01. **f** COX2 mRNA levels in primary CRC cells, HT29 cells and MC38 responding to the incubation with IL-33 (100 ng/mL) or/ and BAY11–7082 (10 μM). Three parallel wells were set for each treatment. Each experiment was performed three times. Data expressed as mean ± SEM. * *P* < 0.05. ** *P* < 0.01. **g** COX2 protein levels in primary CRC cells, HT29 cells and MC38 responding to the incubation with IL-33 (100 ng/mL) or/ and BAY11–7082 (10 μM). Each experiment was performed three times. **h** The knocking-down efficiency of NF-κB P65 in HT29 cells. The P65 mRNA (left panel) and protein (right panel) were both detected. Data expressed as mean ± SEM. ** *P* < 0.01. **i** COX2 mRNA (left panel) and protein (right panel) levels responding to IL-33 incubation (100 ng/mL) for 24 h in HT29 cells transfected with short hairpin RNA expressing plasmid against NF-κB P65 (shP65) or nonsense RNA expressing plasmid (shNC). Each experiment was performed three times. Data expressed as mean ± SEM. ** *P* < 0.01

When ST2 was blocked, IL-33 did not upregulate the mRNA and protein levels of COX2 in primary CRC cells or HT-29 cells anymore (Fig. 4a, b). As shown by flow cytometry assay, 12.3% of primary CRC cells were obviously ST2 positive (Fig. 4c). To further verify COX2 induction was dependent on ST2, we sorted the ST2-positive subset and the ST2-negative subset from primary CRC cells (Fig. 4c). The folds of COX2 induction caused by IL-33 were markedly higher in ST2-positive CRC cells than in ST2-negative CRC cells (Fig. 4d). By analyzing TCGA data, we found that COX2 levels in human CRC were positively correlated with ST2 expression (Fig. 4e). These demonstrate that IL-33 induces COX2/PGE_2_ via ST2-mediated signaling.

Previous studies have revealed the COX2 gene activation relies on assistance of several transcriptional factors including NF-κB, NF-AT, C/EBP and CREB [37–39]. IL-33/ST2 axis could directly stimulate NF-κB nuclear factors in different cell types [40]. Thus, we checked whether the NF-κB signaling mediated the process where IL-33/ST2 axis induced COX2. As expected, the NF-κB specific inhibitor blocked the induction of COX2 mRNA and protein caused by IL-33 in primary CRC cells, HT29 cells and MC38 cells (Fig. 4f, g). In line with this, the NF-κB specific shRNA (shP65) repressed COX2 mRNA and protein levels induced by IL-33 (Fig. 4h, i). Therefore, we concluded that IL-33/ST2 axis induces COX2 through NF-κB signaling.

## Discussion

Previous studies have indicated that IL-33 could regulate proliferation of some types of cells directly or indirectly. IL-33 upregulated CCL2/CCR2 by activating NF-κB and ERK1/2 to facilitate proliferation of decidual sromal cells [41]. Macrophage-derived IL-33 at the maternal-fetal interface promoted trophoblast cells proliferation by activating AKT and ERK1/2 signaling [42]. IL-33 promoted epidermal proliferation to influence wound healing process [43]. IL-33 also induced proliferation of myeloid lineage cells [44] and pancreatic myofibroblasts [7]. The IL-33-triggering signals in cancer cells are poorly understood because cancer cells do not always express its unique receptor ST2. Our previous work revealed that ST2 was expressed in primary CRC cells and HT-29 cells [4]. IL-33 is positive correlated with the CRC proliferation both in human data and in animal experiments (Fig. 1a, b). IL-33 may regulate tumor growth by affecting stromal cells or immune responses [17, 18, 27, 28]. The GSEA showed low degree but statistically significant correlation (Fig. 1a, NES = 1.03, *P* = 0.03) between IL-33 and proliferation regulation gene sets. IL-33 exerts multifunction in cancer progression besides cell proliferation such as immunomodulatory functions by producing chemokines, promoting inflammatory responses, driving Th2-type immune responses, and enhancing cancer stem-like properties [45–47]. Here, we highlight that the IL-33/ST2 axis in CRC cells accelerates proliferation.

By screening the proliferation associated signals, we found that the COX2 inhibitor celecoxib blocked IL-33-induced CRC proliferation. The other six inhibitors also partly impaired the induction-folds of Ki67 and PCNA. Previous studies have revealed that COX2 expression can be reduced by the JNK inhibitor SP600125 [48], the ERK/MAPK inhibitor SB203589 [49], and MEK1/2 inhibitor PD98059 [50]. DNA methyltransferase 5-Aza and the histone methyltransferase inhibitor BIX02189 also regulated COX2 expression [51, 52]. The inhibitor SC-560 could reduce total PGE_2_ production through inhibiting COX1, although it did not  inhibit COX2 at the used concentration [53]. Therefore, we speculated that these six inhibitors may modulate the induction folds of Ki67 and PCNA through regulating COX2 and PGE_2_. However, we think that there may be other possibilities to explain the effects of these inhibitors. DNA methyltransferase, JNK, ERK and MAPK are involved in the mechanism of COX2 induced proliferation [54–56]; so it is reasonable that the inhibitors of these signals partly impair the effect of COX2 on Ki67 and PCNA expression. Therefore, we hypothesized that COX2/PGE_2_ dominantly mediated IL-33-induced CRC proliferation and performed the following experiments.

It is well known that COX2, a key enzyme for PGE_2_ synthesis, can be effectively inhibited by the FDA-approved drug celecoxib [57, 58]. We have circumspectly selected appropriate dosages of SC-560 and celecoxib to inhibit COX1 and COX2, respectively, as their selectivity depends on the concentrations [59–61]. COX2 expression and PGE_2_ production in CRC cells could be elevated by IL-33. Even though celecoxib exhibited complete blockade effect, it was insufficient to certify the COX2/PGE_2_-dependence of IL-33-induced proliferation. This was due to the pharmacodynamics complexity of celecoxib. Celecoxib is usually used as a selective inhibitor of COX2 to prevent PGE_2_ production, but it also exerts effects via other mechanisms. Celecoxib inhibits interleukin-12 subunit folding and secretion by a COX2-independent mechanism involving chaperones of the endoplasmic reticulum [62]. Celecoxib inhibits proliferation of a head and neck squamous cell carcinoma cell line through ER stress response that was proved as a COX2-independent anticancer mechanism [63]. To exclude these COX2-independent mechanisms, we have further provided evidence. The PGE_2_ neutralization assay well demonstrated that PGE_2_ mediated the IL-33-induced proliferation. Therefore, we report that  IL-33 facilitates proliferation of CRC cells by a COX2/PGE_2_-dependent mechanism.

COX2 and PGE_2_ exert critical roles in promoting CRC progression [33, 64]. The mechanism of PGE_2_-induced CRC proliferation has been well described. The receptor EP2 signaled by PGE_2_ promotes CRC proliferation through a Gs-axin-beta-catenin signaling axis [65]. PGE_2_ combines the other receptor EP4 to stimulate CRC proliferation via phosphatidylinositol 3-kinase/Akt pathway [66]. PGE_2_ also activated Ras-mitogen-activated protein kinase cascade to induce intestinal adenoma growth [34].

The recombinant IL-33 concentration used for in vitro experiments are much higher than detected in vivo concentrations [14]. We consider that the biological activity of recombinant IL-33 protein is poorer than endogenous IL-33. This distinction on IL-33 activity may result from the IL-33 cleavage mechanisms. Evidence revealed that full-length IL-33 can be cleaved into a more bioactive form by many proteases in vivo [67, 68]. The cleaved IL-33 has a 10 to 30-fold higher activity than full-length IL-33 in cellular assays [67, 68]. Because of this, many researchers chose much higher doses of recombinant IL-33 for in vitro experiments than in vivo concentrations [4, 69–71]. Thus, the IL-33 concentrations we used in this study are reasonable.

## Conclusion

**Fig. 5:**
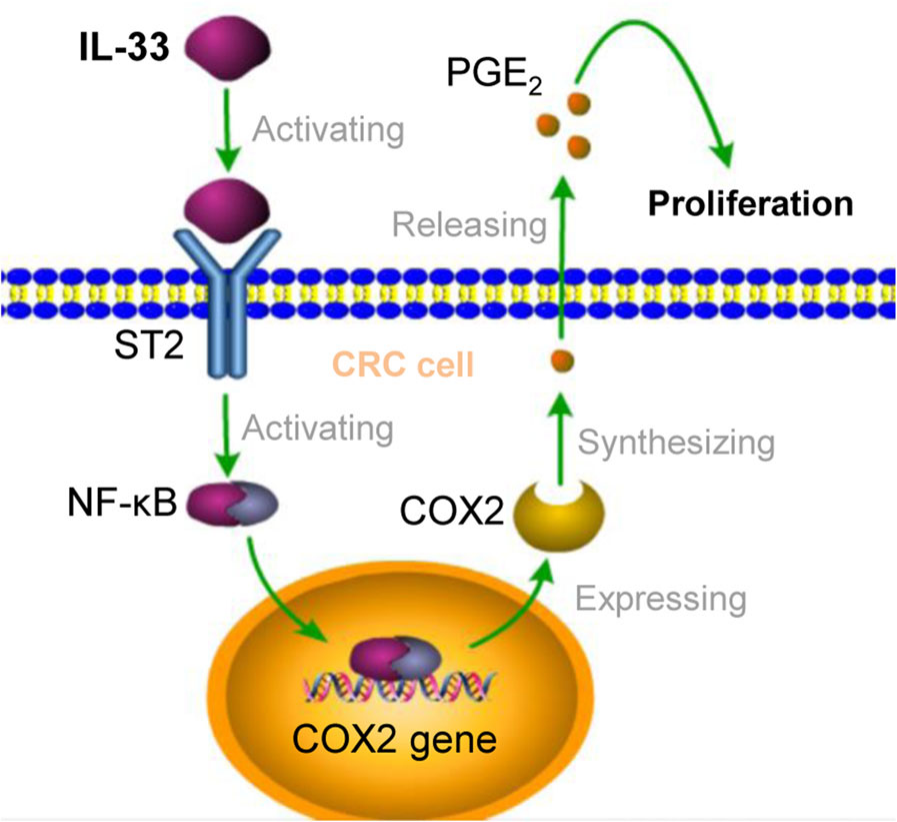
The pathway through which IL-33 stimulates the proliferation signaling of CRC cells

Therefore, we suggest a model to illustrate how IL-33 facilitates CRC proliferation (Fig. 5). IL-33/ST2 axis upregulates COX2 expression through NF-κB signaling, and consequently increases PGE_2_ production; the elevated PGE_2_ mediates the proliferation promoting function of IL-33.

## Additional file

Additional file 1: **Table S1.** Characteristics of the colorectal cancer patients analyzed in this study. **Table S2.** The primers used in this study. **Table S3.** The output of the Gene Set Enrichment Analysis. (DOC 604 kb)

## Abbreviations

COX2: Cycloxygenase-2
CRC: Colorectal cancer
IL-33: Interleukin-33
PCNA: Proliferating cell nuclear antigen
PGE_2_: Prostaglandin E2
