# Correction to: IL-33 facilitates proliferation of colorectal cancer dependent on COX2/PGE_2_

**DOI:** 10.1186/s13046-020-1524-1

**Published:** 2020-01-17

**Authors:** Yongkui Li, Jie Shi, Shanshan Qi, Jian Zhang, Dong Peng, Zhenzhen Chen, Guobin Wang, Zheng Wang, Lin Wang

**Affiliations:** 1 Research Center for Tissue Engineering and Regenerative Medicine, Union Hospital, Tongji Medical College, Huazhong University of Science and Technology, Wuhan, 430022 China; 2 Department of Gastrointestinal Surgery, Union Hospital, Tongji Medical College, Huazhong University of Science and Technology, Wuhan, 430022 China; 3 Department of Clinical Laboratory Union Hospital, Tongji Medical College, Huazhong University of Science and Technology, Wuhan, 430022 China

**Correction to: J Exp Clin Cancer Res**

**https://doi.org/10.1186/s13046-018-0839-7**

In the original publication of this manuscript [1], there are three errors in Fig. 1. The identified errors do not affect the conclusions of the work.

The images of Fig. 1i (rhIL-33 treated group), Fig. 1h (rhIL-33 treated group), and Fig. 1j (rmIL-33 treated group) were mistakenly selected and used. The revised Fig. 1 is shown below.

The authors sincerely apologize for the inconvenience caused to the readers.

**Fig. 1:**
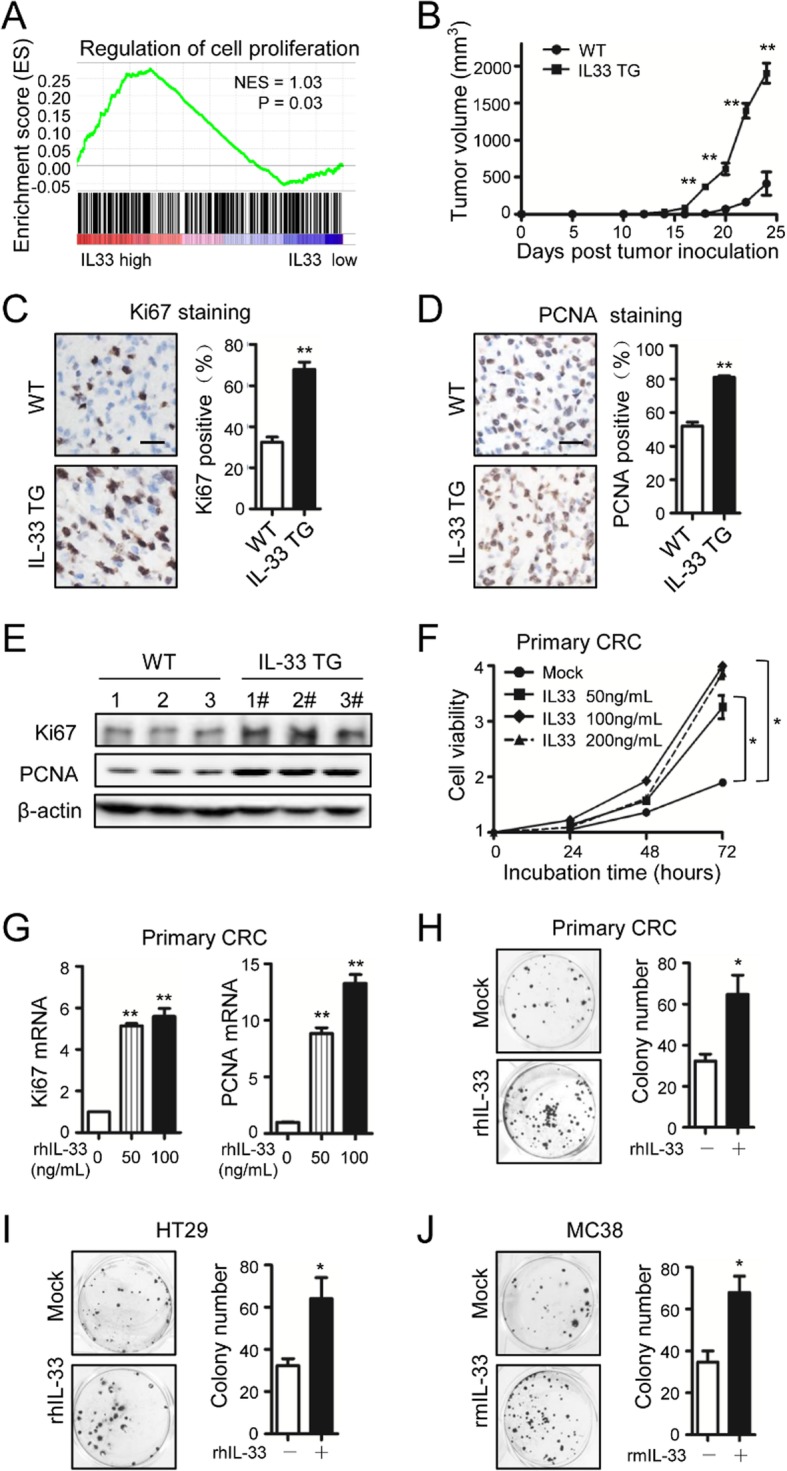
IL-33 promotes CRC proliferation both in vivo and in vitro. **a** Correlation between IL-33 transcripts and the genes involved in the regulation of cell proliferation in CRC. Gene set enrichment analysis was performed using CRC TCGA database. NES = 1.03, *P* = 0.03. **b** Growth curves of MC38 tumors inoculated in IL-33 transgenic mice (IL-33 TG) or wild-type mice (WT). *n* = 7. **c**, **d** Immunohistochemical staining of Ki67 (**c**) and PCNA (**d**) in the MC38 tumors recovered from wild-type and IL-33 transgenic mice at Day 22 post inoculation. The representative images and the statistical proportions of positive cells are shown. Scale bar, 50 μm. *n* = 7. Data expressed as mean ± SEM. **, *P* < 0.01. **e** Western blot of Ki67 and PCNA in the MC38 tumors recovered from wild-type and IL-33 transgenic mice. *n* = 3. **f** Cell viabilities of human primary CRC cells incubated with 0, 50, 100 or 200 ng/mL of rhIL-33 in medium at 24^th^, 48^th^ and 72^nd^ h. Six parallel wells were set for each treatment. The experiment was performed three times. Data expressed as mean ± SEM. * *P* < 0.05. **g** Ki67 and PCNA mRNA levels in primary CRC cells incubated with rhIL-33 (0, 50 or 100 ng/mL) for 24 h. Each experiment was performed three times. Three parallel wells were set for each treatment. Data expressed as mean ± SEM. ** *P* < 0.01. **h**, **i**, **j** The flat colony formation with 500 primary CRC cells (**h**) and 500 HT29 cells (**i**) incubated with rhIL-33 (100 ng/mL) and the flat colony formation with 500 MC38 cells (**j**) incubated with rmIL-33 (100 ng/mL). The number of colony was counted at Day 10. Each experiment was performed three times. Three parallel wells were set for each treatment. The representative images of colonies and the statistical data are shown. Data expressed as mean ± SEM. * *P* < 0.05
