# Supplementary material for: IL-33 facilitates proliferation of colorectal cancer dependent on COX2/PGE2
Source: J Exp Clin Cancer Res. 2018 Aug 17;37:196. doi: 10.1186/s13046-018-0839-7 (PMC6098640; doi:10.1186/s13046-018-0839-7)
Supplement: Supplementary file 1 — Table S1. Characteristics of the colorectal cancer patients analyzed in this study. Table S2. The primers used in this study. Table S3. The output of the Gene Set Enrichment Analysis. (DOC 604 kb) [file 13046_2018_839_MOESM1_ESM.doc]

**Additional file 1**

**Table S1** Characteristics of the colorectal cancer patients analyzed in this study.

|  |  |  | |  | | |
| --- | --- | --- | --- | --- | --- | --- |
|  |  |  | | **Colorectal cancer**  **(cases)** | | **Metastatic colorectal cancer (cases)** |
|  | **Gender** | Female | | 159 | | 29 |
|  |  | Male | | 172 | | 34 |
|  | **T stage** | Tis | | - | | - |
|  |  | T1 | | 9 | | - |
|  |  | T2 | | 69 | | - |
|  |  | T3 | | 232 | | 40 |
|  |  | T4 | | 21 | | 23 |
|  | **N stage** | N0 | | 228 | | 8 |
|  |  | N1 | | 63 | | 25 |
|  |  | N2 | | 39 | | 30 |
|  |  | Nx | | 1 | | - |
|  | **M stage** | M0 | | 331 | | - |
|  |  | M1 | | - | | 63 |
|  | **Histological type** | Adenocarcinoma | | 288 | | 56 |
|  |  | Mucinous Adenocarcinoma | | 42 | | 7 |
|  |  | Unknown | | 1 | | - |
|  | **Localization *** | Distal colon | | 199 | | 28 |
|  |  | Proximal colon | | 130 | | 33 |
|  |  | Unknown | | 2 | | 2 |
|  |  |  |  | |  | |
|  | *Cancers were categorized as proximal (cecum, ascending colon, transverse colon and hepatic flexure), distal (descending colon, sigmoid colon, rectosigmoid and splenic flexure). | | | | | |

**Table S2** The primers used in this study

| **Description and serial number** | | | | **Sequence (5'→3')** |
| --- | --- | --- | --- | --- |
| Mouse Cox2 | Forward | P1 | CCAGCACTTCACCCATCAGT | |
|  | Reverse | P2 | GGGGATACACCTCTCCACCA | |
| Mouse Ki67 | Forward | P3 | ACCTGGTCACCATCAAGCG | |
|  | Reverse | P4 | ACTACAGGCAGCTGGATACG | |
| Mouse PCNA | Forward | P5 | CCTGTGCAAAGAATGGGGTG | |
|  | Reverse | P6 | AGACAGTGGAGTGGCTTTTGT | |
| Mouse Hprt | Forward | P7 | AGCCTAAGATGAGCGCAAGT | |
|  | Reverse | P8 | TTACTAGGCAGATGGCCACA | |
| Human COX2 | Forward | P9 | GCCATGGGGTGGACTTAAATCAT | |
|  | Reverse | P10 | CAGGGACTTGAGGAGGGTAGATCA | |
| Human Ki67 | Forward | P11 | CCCAGGGCAAGAGGCAAATC | |
|  | Reverse | P12 | GCGTCTGGAGCGCAGGGATA | |
| Human PCNA | Forward | P13 | AGGCACTCAAGGACCTCATCAAC | |
|  | Reverse | P14 | TATCCGCGTTATCTTCGGCCCTTAG | |
| Human P65 | Forward | P15 | TGGCCCCTATGTGGAGATCA | |
|  | Reverse | P16 | GTATCTGTGCTCCTCTCGCC | |
| Human GAPDH | Forward | P17 | AAGGCTGTGGGCAAGG | |
|  | Reverse | P18 | TGGAGGAGTGGGTGTCG | |

**Table S3** The output of the Gene Set Enrichment Analysis (GSEA)

| **NAME** | **GENE SYMBOL** | **GENE_TITLE** | **RANK METRIC SCORE** | **RUNNING ES** | **CORE ENRICHMENT** |
| --- | --- | --- | --- | --- | --- |
| row_0 | IL1A | interleukin 1, alpha | 0.309562117 | 0.011334081 | Yes |
| row_1 | EGF | epidermal growth factor (beta-urogastrone) | 0.26502502 | 0.020759022 | Yes |
| row_2 | IL13 | interleukin 13 | 0.203886881 | 0.022453498 | Yes |
| row_3 | NOX1 | NADPH oxidase 1 | 0.193937197 | 0.027446253 | Yes |
| row_4 | TNMD | tenomodulin | 0.187099025 | 0.032179084 | Yes |
| row_5 | TFF1 | trefoil factor 1 (breast cancer, estrogen-inducible sequence expressed in) | 0.178444222 | 0.035069104 | Yes |
| row_6 | ODAM | odontogenic, ameloblast asssociated | 0.178350419 | 0.04184829 | Yes |
| row_7 | IL5 | interleukin 5 (colony-stimulating factor, eosinophil) | 0.172595158 | 0.045597296 | Yes |
| row_8 | EGR3 | early growth response 3 | 0.165233612 | 0.04647133 | Yes |
| row_9 | FGF2 | fibroblast growth factor 2 (basic) | 0.163986996 | 0.051839504 | Yes |
| row_10 | SOX11 | SRY (sex determining region Y)-box 11 | 0.159834146 | 0.054022145 | Yes |
| row_11 | FGFBP1 | fibroblast growth factor binding protein 1 | 0.159399375 | 0.060080994 | Yes |
| row_12 | TGFB2 | transforming growth factor, beta 2 | 0.159228504 | 0.06570082 | Yes |
| row_13 | ATOH8 | atonal homolog 8 (Drosophila) | 0.15874885 | 0.07108615 | Yes |
| row_14 | AGTR2 | angiotensin II receptor, type 2 | 0.157637775 | 0.076429255 | Yes |
| row_15 | UTS2 | urotensin 2 | 0.150582701 | 0.076962665 | Yes |
| row_16 | CXCL3 | chemokine (C-X-C motif) ligand 3 | 0.149420068 | 0.08177714 | Yes |
| row_17 | TDGF1 | teratocarcinoma-derived growth factor 1 | 0.146931693 | 0.08411814 | Yes |
| row_18 | PPBP | pro-platelet basic protein (chemokine (C-X-C motif) ligand 7) | 0.145559117 | 0.08792081 | Yes |
| row_19 | CXCL2 | chemokine (C-X-C motif) ligand 2 | 0.141383111 | 0.08680696 | Yes |
| row_20 | VASH2 | vasohibin 2 | 0.141045287 | 0.09173564 | Yes |
| row_21 | CDH3 | cadherin 3, type 1, P-cadherin (placental) | 0.138516843 | 0.09116163 | Yes |
| row_22 | CXCL11 | chemokine (C-X-C motif) ligand 11 | 0.137367919 | 0.09422042 | Yes |
| row_23 | SERPINE2 | serpin peptidase inhibitor, clade E (nexin, plasminogen activator inhibitor type 1), member 2 | 0.133502901 | 0.09345583 | Yes |
| row_24 | SERPINB3 | serpin peptidase inhibitor, clade B (ovalbumin), member 3 | 0.133200407 | 0.09722126 | Yes |
| row_25 | CSF3 | colony stimulating factor 3 (granulocyte) | 0.131033897 | 0.09939051 | Yes |
| row_26 | BTC | betacellulin | 0.130176723 | 0.1019597 | Yes |
| row_27 | TNFSF9 | tumor necrosis factor (ligand) superfamily, member 9 | 0.128943458 | 0.10513079 | Yes |
| row_28 | IL12B | interleukin 12B (natural killer cell stimulatory factor 2, cytotoxic lymphocyte maturation factor 2, p40) | 0.12819694 | 0.10892231 | Yes |
| row_29 | ADAMTS8 | ADAM metallopeptidase with thrombospondin type 1 motif, 8 | 0.127980858 | 0.1133544 | Yes |
| row_30 | FABP6 | fatty acid binding protein 6, ileal (gastrotropin) | 0.125743583 | 0.113376185 | Yes |
| row_31 | ADIPOQ | adiponectin, C1Q and collagen domain containing | 0.125411898 | 0.11792689 | Yes |
| row_32 | FAP | fibroblast activation protein, alpha | 0.123151071 | 0.11676882 | Yes |
| row_33 | HOXD13 | homeobox D13 | 0.122666575 | 0.12013386 | Yes |
| row_34 | null | null | 0.122546144 | 0.12435938 | Yes |
| row_35 | CXCL1 | chemokine (C-X-C motif) ligand 1 (melanoma growth stimulating activity, alpha) | 0.122292824 | 0.12835899 | Yes |
| row_36 | CER1 | cerberus 1, cysteine knot superfamily, homolog (Xenopus laevis) | 0.121352881 | 0.13124157 | Yes |
| row_37 | KIT | v-kit Hardy-Zuckerman 4 feline sarcoma viral oncogene homolog | 0.120405018 | 0.13408813 | Yes |
| row_38 | SHC4 | SHC (Src homology 2 domain containing) family, member 4 | 0.120391108 | 0.13866425 | Yes |
| row_39 | FABP7 | fatty acid binding protein 7, brain | 0.120277025 | 0.14323604 | Yes |
| row_40 | DRD3 | dopamine receptor D3 | 0.120003983 | 0.14736491 | Yes |
| row_41 | EGR1 | early growth response 1 | 0.119426087 | 0.15125558 | Yes |
| row_42 | LEP | leptin (obesity homolog, mouse) | 0.116975151 | 0.14921397 | Yes |
| row_43 | EYA1 | eyes absent homolog 1 (Drosophila) | 0.114988185 | 0.14861068 | Yes |
| row_44 | FUZ | fuzzy homolog (Drosophila) | 0.11401429 | 0.14970046 | Yes |
| row_45 | NR4A3 | nuclear receptor subfamily 4, group A, member 3 | 0.113469683 | 0.15249966 | Yes |
| row_46 | GDF5 | growth differentiation factor 5 (cartilage-derived morphogenetic protein-1) | 0.113360755 | 0.15637603 | Yes |
| row_47 | TLR4 | toll-like receptor 4 | 0.11298725 | 0.15980569 | Yes |
| row_48 | RETN | resistin | 0.112045996 | 0.16211824 | Yes |
| row_49 | PRKD1 | protein kinase D1 | 0.110495262 | 0.16004659 | Yes |
| row_50 | KBTBD10 | kelch repeat and BTB (POZ) domain containing 10 | 0.109874472 | 0.16227661 | Yes |
| row_51 | null | null | 0.109662808 | 0.16644494 | Yes |
| row_52 | ALDH1A2 | aldehyde dehydrogenase 1 family, member A2 | 0.108962581 | 0.1686403 | Yes |
| row_53 | FGF5 | fibroblast growth factor 5 | 0.108905338 | 0.17234732 | Yes |
| row_54 | FABP1 | fatty acid binding protein 1, liver | 0.108506031 | 0.1751741 | Yes |
| row_55 | WNT2 | wingless-type MMTV integration site family member 2 | 0.107912682 | 0.17776208 | Yes |
| row_56 | EREG | epiregulin | 0.107480153 | 0.18033361 | Yes |
| row_57 | LRG1 | leucine-rich alpha-2-glycoprotein 1 | 0.10733401 | 0.18398091 | Yes |
| row_58 | null | null | 0.107133538 | 0.18740432 | Yes |
| row_59 | SHH | sonic hedgehog homolog (Drosophila) | 0.106986754 | 0.19125468 | Yes |
| row_60 | HBEGF | heparin-binding EGF-like growth factor | 0.106206559 | 0.193129 | Yes |
| row_61 | IL23R | interleukin 23 receptor | 0.105953947 | 0.1969401 | Yes |
| row_62 | null | null | 0.105567522 | 0.19879015 | Yes |
| row_63 | MYCNOS | v-myc myelocytomatosis viral related oncogene, neuroblastoma derived (avian) opposite strand | 0.105485834 | 0.20258345 | Yes |
| row_64 | null | null | 0.104375713 | 0.20179303 | Yes |
| row_65 | WT1 | Wilms tumor 1 | 0.103155315 | 0.20138876 | Yes |
| row_66 | NOV | nephroblastoma overexpressed gene | 0.102049559 | 0.20115872 | Yes |
| row_67 | GAL | galanin | 0.102034084 | 0.20503709 | Yes |
| row_68 | PROK1 | prokineticin 1 | 0.101323947 | 0.20672582 | Yes |
| row_69 | SGK2 | serum/glucocorticoid regulated kinase 2 | 0.101241626 | 0.2103578 | Yes |
| row_70 | CYR61 | cysteine-rich, angiogenic inducer, 61 | 0.100450106 | 0.21136454 | Yes |
| row_71 | FAS | Fas (TNF receptor superfamily, member 6) | 0.099840045 | 0.2140782 | Yes |
| row_72 | MYOCD | myocardin | 0.099729814 | 0.21678767 | Yes |
| row_73 | PRRX1 | paired related homeobox 1 | 0.098106161 | 0.21294752 | Yes |
| row_74 | IL1B | interleukin 1, beta | 0.098081328 | 0.21667564 | Yes |
| row_75 | AREG | amphiregulin (schwannoma-derived growth factor) | 0.097818807 | 0.2190962 | Yes |
| row_76 | KCNA5 | potassium voltage-gated channel, shaker-related subfamily, member 5 | 0.097666524 | 0.2219435 | Yes |
| row_77 | LIF | leukemia inhibitory factor (cholinergic differentiation factor) | 0.097280048 | 0.22455984 | Yes |
| row_78 | ACE2 | angiotensin I converting enzyme (peptidyl-dipeptidase A) 2 | 0.096865579 | 0.22759297 | Yes |
| row_79 | NOS1 | nitric oxide synthase 1 (neuronal) | 0.09664271 | 0.23018509 | Yes |
| row_80 | CGA | glycoprotein hormones, alpha polypeptide | 0.096159659 | 0.23275885 | Yes |
| row_81 | NR4A1 | nuclear receptor subfamily 4, group A, member 1 | 0.095707275 | 0.23445037 | Yes |
| row_82 | EIF5A2 | eukaryotic translation initiation factor 5A2 | 0.095686033 | 0.23808743 | Yes |
| row_83 | ETV4 | ets variant gene 4 (E1A enhancer binding protein, E1AF) | 0.095427155 | 0.24063335 | Yes |
| row_84 | IL6 | interleukin 6 (interferon, beta 2) | 0.095187761 | 0.24403523 | Yes |
| row_85 | LHX5 | LIM homeobox 5 | 0.093906902 | 0.24284689 | Yes |
| row_86 | FZD3 | frizzled homolog 3 (Drosophila) | 0.093746796 | 0.2455452 | Yes |
| row_87 | NTRK2 | neurotrophic tyrosine kinase, receptor, type 2 | 0.093315065 | 0.24801083 | Yes |
| row_88 | HMGA2 | high mobility group AT-hook 2 | 0.091084532 | 0.24779652 | Yes |
| row_89 | CLCF1 | cardiotrophin-like cytokine factor 1 | 0.091074988 | 0.25125834 | Yes |
| row_90 | GATA2 | GATA binding protein 2 | 0.090593137 | 0.2536205 | Yes |
| row_91 | PPARGC1A | peroxisome proliferative activated receptor, gamma, coactivator 1, alpha | 0.089285448 | 0.25550044 | Yes |
| row_92 | ID4 | inhibitor of DNA binding 4, dominant negative helix-loop-helix protein | 0.088523418 | 0.25800022 | Yes |
| row_93 | IRS1 | insulin receptor substrate 1 | 0.088414758 | 0.26114464 | Yes |
| row_94 | WFDC1 | WAP four-disulfide core domain 1 | 0.086238019 | 0.26290876 | Yes |
| row_95 | ANXA1 | annexin A1 | 0.084034972 | 0.2611289 | Yes |
| row_96 | ECM1 | extracellular matrix protein 1 | 0.082552321 | 0.26210412 | Yes |
| row_97 | PAX6 | paired box gene 6 (aniridia, keratitis) | 0.078583471 | 0.26098213 | Yes |
| row_98 | null | null | 0.074316926 | 0.2560215 | Yes |
| row_99 | ATF3 | activating transcription factor 3 | 0.074108005 | 0.2579733 | Yes |
| row_100 | EEF1E1 | eukaryotic translation elongation factor 1 epsilon 1 | 0.072859511 | 0.25966144 | Yes |
| row_101 | SPRY1 | sprouty homolog 1, antagonist of FGF signaling (Drosophila) | 0.072192729 | 0.2606754 | Yes |
| row_102 | IHH | Indian hedgehog homolog (Drosophila) | 0.072169945 | 0.2634186 | Yes |
| row_103 | ST6GAL1 | ST6 beta-galactosamide alpha-2,6-sialyltranferase 1 | 0.070083484 | 0.26197353 | Yes |
| row_104 | S100A11 | S100 calcium binding protein A11 | 0.068073526 | 0.2593707 | Yes |
| row_105 | ERRFI1 | ERBB receptor feedback inhibitor 1 | 0.0667293 | 0.25844693 | Yes |
| row_106 | SLC39A10 | solute carrier family 39 (zinc transporter), member 10 | 0.065455414 | 0.25898856 | Yes |
| row_107 | PTBP2 | polypyrimidine tract binding protein 2 | 0.06508854 | 0.25994876 | Yes |
| row_108 | ZNF503 | zinc finger protein 503 | 0.064566433 | 0.2608891 | Yes |
| row_109 | TPM1 | tropomyosin 1 (alpha) | 0.06374447 | 0.2602844 | Yes |
| row_110 | NMB | neuromedin B | 0.063643143 | 0.26248723 | Yes |
| row_111 | NOS3 | nitric oxide synthase 3 (endothelial cell) | 0.063608497 | 0.26490504 | Yes |
| row_112 | STS | steroid sulfatase (microsomal), arylsulfatase C, isozyme S | 0.063406609 | 0.26666635 | Yes |
| row_113 | RARG | retinoic acid receptor, gamma | 0.062866479 | 0.26797464 | Yes |
| row_114 | SMYD2 | SET and MYND domain containing 2 | 0.06219817 | 0.26795992 | Yes |
| row_115 | TP53 | tumor protein p53 (Li-Fraumeni syndrome) | 0.061015237 | 0.2646563 | Yes |
| row_116 | CDKN1A | cyclin-dependent kinase inhibitor 1A (p21, Cip1) | 0.06086874 | 0.26653743 | Yes |
| row_117 | S100A13 | S100 calcium binding protein A13 | 0.060800061 | 0.26884848 | Yes |
| row_118 | IFT74 | intraflagellar transport 74 homolog (Chlamydomonas) | 0.059095554 | 0.26439056 | Yes |
| row_119 | CDKN3 | cyclin-dependent kinase inhibitor 3 (CDK2-associated dual specificity phosphatase) | 0.058465336 | 0.26509902 | Yes |
| row_120 | FXN | frataxin | 0.057922587 | 0.26492178 | Yes |
| row_121 | ID2 | inhibitor of DNA binding 2, dominant negative helix-loop-helix protein | 0.057657894 | 0.26646462 | Yes |
| row_122 | MNAT1 | menage a trois homolog 1, cyclin H assembly factor (Xenopus laevis) | 0.057232939 | 0.26712623 | Yes |
| row_123 | TIMP1 | TIMP metallopeptidase inhibitor 1 | 0.056812759 | 0.26777187 | Yes |
| row_124 | NFIB | nuclear factor I/B | 0.0555434 | 0.26663914 | Yes |
| row_125 | CCNB1 | cyclin B1 | 0.055211809 | 0.2674402 | Yes |
| row_126 | NPM1 | nucleophosmin (nucleolar phosphoprotein B23, numatrin) | 0.055208854 | 0.2695387 | Yes |
| row_127 | JAG1 | jagged 1 (Alagille syndrome) | 0.054679602 | 0.27031952 | Yes |
| row_128 | RBBP9 | retinoblastoma binding protein 9 | 0.053829577 | 0.2689054 | Yes |
| row_129 | PDCD5 | programmed cell death 5 | 0.053822897 | 0.27095124 | Yes |
| row_130 | NDUFS4 | NADH dehydrogenase (ubiquinone) Fe-S protein 4, 18kDa (NADH-coenzyme Q reductase) | 0.053293165 | 0.27124685 | Yes |
| row_131 | ADK | adenosine kinase | 0.053159602 | 0.27261868 | Yes |
| row_132 | MED31 | mediator of RNA polymerase II transcription, subunit 31 homolog (S. cerevisiae) | 0.05237833 | 0.2717982 | Yes |
| row_133 | JUNB | jun B proto-oncogene | 0.052257027 | 0.27356824 | Yes |
| row_134 | ITGA2 | integrin, alpha 2 (CD49B, alpha 2 subunit of VLA-2 receptor) | 0.051764093 | 0.2731569 | Yes |
| row_135 | STXBP4 | syntaxin binding protein 4 | 0.051752601 | 0.27512407 | Yes |
| row_136 | CD24 | CD24 molecule | 0.051623639 | 0.27687004 | Yes |
| row_137 | ITGA1 | integrin, alpha 1 | 0.05141893 | 0.2781757 | Yes |
| row_138 | BTG3 | BTG family, member 3 | 0.050043114 | 0.27272493 | No |
| row_139 | S100A6 | S100 calcium binding protein A6 | 0.048294365 | 0.26677516 | No |
| row_140 | PINX1 | - | 0.047106188 | 0.26186153 | No |
| row_141 | MDM2 | Mdm2, transformed 3T3 cell double minute 2, p53 binding protein (mouse) | 0.046364721 | 0.26124498 | No |
| row_142 | LAMB1 | laminin, beta 1 | 0.044957593 | 0.25322202 | No |
| row_143 | IFITM1 | interferon induced transmembrane protein 1 (9-27) | 0.044481028 | 0.2512363 | No |
| row_144 | CEBPA | CCAAT/enhancer binding protein (C/EBP), alpha | 0.044149581 | 0.24988675 | No |
| row_145 | null | null | 0.044104859 | 0.25134695 | No |
| row_146 | WDR77 | WD repeat domain 77 | 0.043414656 | 0.24888815 | No |
| row_147 | NDRG2 | NDRG family member 2 | 0.042962443 | 0.24770977 | No |
| row_148 | GLMN | glomulin, FKBP associated protein | 0.042445041 | 0.24348402 | No |
| row_149 | IGFBP7 | insulin-like growth factor binding protein 7 | 0.041157644 | 0.23747924 | No |
| row_150 | TGFBR2 | transforming growth factor, beta receptor II (70/80kDa) | 0.039964471 | 0.2331592 | No |
| row_151 | BAX | BCL2-associated X protein | 0.03979513 | 0.23207667 | No |
| row_152 | CNOT8 | CCR4-NOT transcription complex, subunit 8 | 0.039305348 | 0.2288129 | No |
| row_153 | null | null | 0.038588189 | 0.22508936 | No |
| row_154 | NAP1L1 | nucleosome assembly protein 1-like 1 | 0.038541637 | 0.22655433 | No |
| row_155 | null | null | 0.037775908 | 0.22236738 | No |
| row_156 | null | null | 0.037662622 | 0.22185259 | No |
| row_157 | HMGCR | 3-hydroxy-3-methylglutaryl-Coenzyme A reductase | 0.037606522 | 0.22328202 | No |
| row_158 | GLUL | glutamate-ammonia ligase (glutamine synthetase) | 0.036864586 | 0.21711406 | No |
| row_159 | BTG2 | BTG family, member 2 | 0.036698256 | 0.21764393 | No |
| row_160 | CTSH | cathepsin H | 0.036679316 | 0.21903813 | No |
| row_161 | NME1 | non-metastatic cells 1, protein (NM23A) expressed in | 0.035914537 | 0.21348284 | No |
| row_162 | RAB33B | RAB33B, member RAS oncogene family | 0.035318859 | 0.20963502 | No |
| row_163 | KLF11 | Kruppel-like factor 11 | 0.034781307 | 0.20641555 | No |
| row_164 | LGALS3 | lectin, galactoside-binding, soluble, 3 (galectin 3) | 0.032442499 | 0.18299472 | No |
| row_165 | BMPR1A | bone morphogenetic protein receptor, type IA | 0.032129746 | 0.1824859 | No |
| row_166 | null | null | 0.031876266 | 0.18131863 | No |
| row_167 | B4GALT7 | xylosylprotein beta 1,4-galactosyltransferase, polypeptide 7 (galactosyltransferase I) | 0.031509727 | 0.1786236 | No |
| row_168 | CDC73 | cell division cycle 73, Paf1/RNA polymerase II complex component, homolog (S. cerevisiae) | 0.031335656 | 0.17808458 | No |
| row_169 | KRAS | v-Ki-ras2 Kirsten rat sarcoma viral oncogene homolog | 0.030828822 | 0.17363356 | No |
| row_170 | ITGB1BP1 | integrin beta 1 binding protein 1 | 0.030824011 | 0.1748052 | No |
| row_171 | PRDX3 | peroxiredoxin 3 | 0.030313089 | 0.1701183 | No |
| row_172 | COPS8 | COP9 constitutive photomorphogenic homolog subunit 8 (Arabidopsis) | 0.029863678 | 0.16671193 | No |
| row_173 | LAMC1 | laminin, gamma 1 (formerly LAMB2) | 0.029698581 | 0.16611068 | No |
| row_174 | CDK4 | cyclin-dependent kinase 4 | 0.029669158 | 0.16723841 | No |
| row_175 | RNF41 | ring finger protein 41 | 0.028604863 | 0.15945892 | No |
| row_176 | ILK | integrin-linked kinase | 0.028142639 | 0.15468954 | No |
| row_177 | null | null | 0.02786066 | 0.15336964 | No |
| row_178 | PGGT1B | protein geranylgeranyltransferase type I, beta subunit | 0.027780304 | 0.15356052 | No |
| row_179 | CD46 | CD46 molecule, complement regulatory protein | 0.027724881 | 0.15461436 | No |
| row_180 | PDCL3 | phosducin-like 3 | 0.026845774 | 0.14720052 | No |
| row_181 | NDFIP1 | Nedd4 family interacting protein 1 | 0.026789922 | 0.14800256 | No |
| row_182 | PHB2 | prohibitin 2 | 0.02669302 | 0.14815213 | No |
| row_183 | TGFBR1 | transforming growth factor, beta receptor I (activin A receptor type II-like kinase, 53kDa) | 0.026385475 | 0.14634363 | No |
| row_184 | SRF | serum response factor (c-fos serum response element-binding transcription factor) | 0.026206573 | 0.14604218 | No |
| row_185 | CD59 | CD59 molecule, complement regulatory protein | 0.026133634 | 0.14638674 | No |
| row_186 | ARHGEF2 | rho/rac guanine nucleotide exchange factor (GEF) 2 | 0.026061205 | 0.14607976 | No |
| row_187 | GNL3 | guanine nucleotide binding protein-like 3 (nucleolar) | 0.025429608 | 0.13861209 | No |
| row_188 | PDCD10 | programmed cell death 10 | 0.024755439 | 0.1328489 | No |
| row_189 | PRKRA | protein kinase, interferon-inducible double stranded RNA dependent activator | 0.024493508 | 0.13204981 | No |
| row_190 | HDAC2 | histone deacetylase 2 | 0.024373461 | 0.13189495 | No |
| row_191 | YES1 | v-yes-1 Yamaguchi sarcoma viral oncogene homolog 1 | 0.02370658 | 0.12457805 | No |
| row_192 | PAWR | PRKC, apoptosis, WT1, regulator | 0.023655534 | 0.12526095 | No |
| row_193 | STRAP | serine/threonine kinase receptor associated protein | 0.023513548 | 0.12464087 | No |
| row_194 | DDX20 | DEAD (Asp-Glu-Ala-Asp) box polypeptide 20 | 0.023042431 | 0.12227278 | No |
| row_195 | NACA | nascent-polypeptide-associated complex alpha polypeptide | 0.022988832 | 0.12293033 | No |
| row_196 | PDCD2 | programmed cell death 2 | 0.022383833 | 0.11923963 | No |
| row_197 | RPS15A | ribosomal protein S15a | 0.022257224 | 0.119220585 | No |
| row_198 | CD81 | CD81 molecule | 0.020849403 | 0.107037306 | No |
| row_199 | JTB | jumping translocation breakpoint | 0.020583959 | 0.106738396 | No |
| row_200 | TRIM32 | tripartite motif-containing 32 | 0.020556172 | 0.107303485 | No |
| row_201 | RPS4X | ribosomal protein S4, X-linked | 0.020387147 | 0.10656457 | No |
| row_202 | TOPORS | topoisomerase I binding, arginine/serine-rich | 0.020210532 | 0.104737625 | No |
| row_203 | CNOT6 | CCR4-NOT transcription complex, subunit 6 | 0.02005562 | 0.103769846 | No |
| row_204 | GNG5 | guanine nucleotide binding protein (G protein), gamma 5 | 0.019336389 | 0.10039583 | No |
| row_205 | ITGB1 | integrin, beta 1 (fibronectin receptor, beta polypeptide, antigen CD29 includes MDF2, MSK12) | 0.01911561 | 0.100041114 | No |
| row_206 | PPP2R5C | protein phosphatase 2, regulatory subunit B (B56), gamma isoform | 0.018867629 | 0.09859565 | No |
| row_207 | PRKAA1 | protein kinase, AMP-activated, alpha 1 catalytic subunit | 0.018217217 | 0.094962835 | No |
| row_208 | XRCC5 | X-ray repair complementing defective repair in Chinese hamster cells 5 (double-strand-break rejoining; Ku autoantigen, 80kDa) | 0.018165825 | 0.0952208 | No |
| row_209 | null | null | 0.016341154 | 0.089570306 | No |
| row_210 | CUL2 | cullin 2 | 0.015781084 | 0.08865631 | No |
| row_211 | DNAJA2 | DnaJ (Hsp40) homolog, subfamily A, member 2 | 0.014783117 | 0.08683933 | No |
| row_212 | CNBP | CCHC-type zinc finger, nucleic acid binding protein | 0.012034979 | 0.08426911 | No |
| row_213 | RNF10 | ring finger protein 10 | -0.012139651 | 0.08451428 | No |
| row_214 | CTBP1 | C-terminal binding protein 1 | -0.013853165 | 0.083527006 | No |
| row_215 | STAMBP | STAM binding protein | -0.015040683 | 0.082584865 | No |
| row_216 | EIF2AK1 | eukaryotic translation initiation factor 2-alpha kinase 1 | -0.016748618 | 0.07911249 | No |
| row_217 | BECN1 | beclin 1 (coiled-coil, myosin-like BCL2 interacting protein) | -0.016786909 | 0.079750575 | No |
| row_218 | RELA | v-rel reticuloendotheliosis viral oncogene homolog A, nuclear factor of kappa light polypeptide gene enhancer in B-cells 3, p65 (avian) | -0.017201405 | 0.07889056 | No |
| row_219 | TBRG1 | transforming growth factor beta regulator 1 | -0.018275576 | 0.07504371 | No |
| row_220 | TSC2 | tuberous sclerosis 2 | -0.019009341 | 0.07230605 | No |
| row_221 | null | null | -0.0192197 | 0.072171554 | No |
| row_222 | SOS1 | son of sevenless homolog 1 (Drosophila) | -0.020352716 | 0.06861991 | No |
| row_223 | null | null | -0.021058738 | 0.066176414 | No |
| row_224 | null | null | -0.021408629 | 0.06526007 | No |
| row_225 | DGCR8 | DiGeorge syndrome critical region gene 8 | -0.021758515 | 0.06349196 | No |
| row_226 | HP1BP3 | heterochromatin protein 1, binding protein 3 | -0.022073023 | 0.061952073 | No |
| row_227 | PTPN6 | protein tyrosine phosphatase, non-receptor type 6 | -0.022558635 | 0.058268018 | No |
| row_228 | TMEM115 | transmembrane protein 115 | -0.022746503 | 0.056321204 | No |
| row_229 | RAPGEF1 | Rap guanine nucleotide exchange factor (GEF) 1 | -0.022769585 | 0.05675416 | No |
| row_230 | FZR1 | fizzy/cell division cycle 20 related 1 (Drosophila) | -0.023428548 | 0.052670643 | No |
| row_231 | MAFG | v-maf musculoaponeurotic fibrosarcoma oncogene homolog G (avian) | -0.023486273 | 0.052698314 | No |
| row_232 | C2ORF18 | chromosome 2 open reading frame 18 | -0.02350167 | 0.053591624 | No |
| row_233 | PAK1 | p21/Cdc42/Rac1-activated kinase 1 (STE20 homolog, yeast) | -0.024047526 | 0.048882846 | No |
| row_234 | BAP1 | BRCA1 associated protein-1 (ubiquitin carboxy-terminal hydrolase) | -0.02418972 | 0.04915352 | No |
| row_235 | CAPN1 | calpain 1, (mu/I) large subunit | -0.024451314 | 0.04748777 | No |
| row_236 | TAF6 | TAF6 RNA polymerase II, TATA box binding protein (TBP)-associated factor, 80kDa | -0.024792815 | 0.046916317 | No |
| row_237 | NUDT16 | nudix (nucleoside diphosphate linked moiety X)-type motif 16 | -0.025449906 | 0.04226084 | No |
| row_238 | HLA-A | major histocompatibility complex, class I, A | -0.025471255 | 0.043229017 | No |
| row_239 | MXD4 | MAX dimerization protein 4 | -0.025920974 | 0.038807712 | No |
| row_240 | SSBP3 | single stranded DNA binding protein 3 | -0.025943363 | 0.03892878 | No |
| row_241 | IKBKB | inhibitor of kappa light polypeptide gene enhancer in B-cells, kinase beta | -0.026202636 | 0.037113335 | No |
| row_242 | RXRA | retinoid X receptor, alpha | -0.026423153 | 0.03638759 | No |
| row_243 | null | null | -0.02657114 | 0.03501868 | No |
| row_244 | RREB1 | ras responsive element binding protein 1 | -0.026604015 | 0.03602991 | No |
| row_245 | BAD | BCL2-antagonist of cell death | -0.027691202 | 0.026269317 | No |
| row_246 | CTNNBIP1 | catenin, beta interacting protein 1 | -0.027724773 | 0.02732315 | No |
| row_247 | MFN2 | mitofusin 2 | -0.029273504 | 0.01610886 | No |
| row_248 | HSF1 | heat shock transcription factor 1 | -0.029769475 | 0.012266362 | No |
| row_249 | PPARD | peroxisome proliferative activated receptor, delta | -0.029818406 | 0.012750985 | No |
| row_250 | CSK | c-src tyrosine kinase | -0.029944669 | 0.013456671 | No |
| row_251 | null | null | -0.030103499 | 0.012870816 | No |
| row_252 | ITCH | itchy homolog E3 ubiquitin protein ligase (mouse) | -0.030253105 | 0.01250691 | No |
| row_253 | DVL2 | dishevelled, dsh homolog 2 (Drosophila) | -0.030319614 | 0.013443111 | No |
| row_254 | STK4 | serine/threonine kinase 4 | -0.030356796 | 0.014596987 | No |
| row_255 | NOTCH1 | Notch homolog 1, translocation-associated (Drosophila) | -0.030524919 | 0.013378361 | No |
| row_256 | CRKL | v-crk sarcoma virus CT10 oncogene homolog (avian)-like | -0.030526076 | 0.014538672 | No |
| row_257 | HGS | hepatocyte growth factor-regulated tyrosine kinase substrate | -0.030683365 | 0.014407384 | No |
| row_258 | ZNF335 | zinc finger protein 335 | -0.031151008 | 0.009968611 | No |
| row_259 | SPHK2 | sphingosine kinase 2 | -0.031500187 | 0.008138267 | No |
| row_260 | PARP10 | poly (ADP-ribose) polymerase family, member 10 | -0.032400548 | 0.002233149 | No |
| row_261 | PPP1R9B | protein phosphatase 1, regulatory subunit 9B, spinophilin | -0.032528412 | 0.002604518 | No |
| row_262 | FTH1 | ferritin, heavy polypeptide 1 | -0.033182941 | -0.002405809 | No |
| row_263 | ATF2 | activating transcription factor 2 | -0.033250578 | -0.001574464 | No |
| row_264 | PER2 | period homolog 2 (Drosophila) | -0.033251066 | -3.11E-04 | No |
| row_265 | TNFRSF1B | tumor necrosis factor receptor superfamily, member 1B | -0.034141239 | -0.005717002 | No |
| row_266 | MLL2 | myeloid/lymphoid or mixed-lineage leukemia 2 | -0.034179445 | -0.005282877 | No |
| row_267 | TMEM127 | transmembrane protein 127 | -0.034379195 | -0.00570621 | No |
| row_268 | TNFRSF14 | tumor necrosis factor receptor superfamily, member 14 (herpesvirus entry mediator) | -0.034743451 | -0.007845802 | No |
| row_269 | IFT122 | intraflagellar transport 122 homolog (Chlamydomonas) | -0.035083298 | -0.009323688 | No |
| row_270 | JUP | junction plakoglobin | -0.035423703 | -0.011004898 | No |
| row_271 | SRC | v-src sarcoma (Schmidt-Ruppin A-2) viral oncogene homolog (avian) | -0.035443898 | -0.009873921 | No |
| row_272 | null | null | -0.035469703 | -0.0085257 | No |
| row_273 | RARA | retinoic acid receptor, alpha | -0.035746749 | -0.009762105 | No |
| row_274 | GATA6 | GATA binding protein 6 | -0.035932004 | -0.009910152 | No |
| row_275 | HLA-E | major histocompatibility complex, class I, E | -0.036205426 | -0.010696597 | No |
| row_276 | FOSL2 | FOS-like antigen 2 | -0.036711205 | -0.012761394 | No |
| row_277 | PRKD2 | protein kinase D2 | -0.036847956 | -0.012009573 | No |
| row_278 | null | null | -0.03691747 | -0.010822585 | No |
| row_279 | FLCN | folliculin | -0.037388518 | -0.011564059 | No |
| row_280 | null | null | -0.037443303 | -0.011222136 | No |
| row_281 | TICAM1 | toll-like receptor adaptor molecule 1 | -0.038940985 | -0.021420171 | No |
| row_282 | NCK2 | NCK adaptor protein 2 | -0.039391212 | -0.02273431 | No |
| row_283 | MEN1 | multiple endocrine neoplasia I | -0.040598463 | -0.028327823 | No |
| row_284 | ICOSLG | inducible T-cell co-stimulator ligand | -0.040946525 | -0.02936658 | No |
| row_285 | null | null | -0.041217364 | -0.03082757 | No |
| row_286 | DDR1 | discoidin domain receptor family, member 1 | -0.041246518 | -0.029476034 | No |
| row_287 | BCL2L1 | BCL2-like 1 | -0.041763306 | -0.031781323 | No |
| row_288 | LRP5 | low density lipoprotein receptor-related protein 5 | -0.042492621 | -0.032761313 | No |
| row_289 | SMAD3 | SMAD, mothers against DPP homolog 3 (Drosophila) | -0.044374298 | -0.0423203 | No |
| row_290 | ETS1 | v-ets erythroblastosis virus E26 oncogene homolog 1 (avian) | -0.045475356 | -0.04707965 | No |
| row_291 | ERBB3 | v-erb-b2 erythroblastic leukemia viral oncogene homolog 3 (avian) | -0.0463599 | -0.048128907 | No |
| row_292 | MXI1 | MAX interactor 1 | -0.047322471 | -0.051520467 | No |
| row_293 | MAD1L1 | MAD1 mitotic arrest deficient-like 1 (yeast) | -0.048020914 | -0.053155378 | No |
| row_294 | IL28RA | interleukin 28 receptor, alpha (interferon, lambda receptor) | -0.048046347 | -0.05176164 | No |
| row_295 | PTK2B | PTK2B protein tyrosine kinase 2 beta | -0.048208788 | -0.050361723 | No |
| row_296 | NDRG1 | N-myc downstream regulated gene 1 | -0.048608582 | -0.050460454 | No |
| row_297 | SPINT1 | serine peptidase inhibitor, Kunitz type 1 | -0.048946213 | -0.049897563 | No |
| row_298 | TNK1 | tyrosine kinase, non-receptor, 1 | -0.049198072 | -0.049325097 | No |
| row_299 | PTK6 | PTK6 protein tyrosine kinase 6 | -0.049635991 | -0.049168512 | No |
| row_300 | PTPRJ | protein tyrosine phosphatase, receptor type, J | -0.050021879 | -0.04964605 | No |
| row_301 | C1ORF172 | chromosome 1 open reading frame 172 | -0.050052881 | -0.047743518 | No |
| row_302 | BTN2A2 | butyrophilin, subfamily 2, member A2 | -0.050415762 | -0.04669224 | No |
| row_303 | C10ORF54 | chromosome 10 open reading frame 54 | -0.050543766 | -0.045419835 | No |
| row_304 | NEU1 | sialidase 1 (lysosomal sialidase) | -0.052039273 | -0.049064636 | No |
| row_305 | TNS3 | tensin 3 | -0.05213486 | -0.047948018 | No |
| row_306 | POR | P450 (cytochrome) oxidoreductase | -0.052465737 | -0.047251347 | No |
| row_307 | ERBB2 | v-erb-b2 erythroblastic leukemia viral oncogene homolog 2, neuro/glioblastoma derived oncogene homolog (avian) | -0.052509669 | -0.04547169 | No |
| row_308 | PYCARD | PYD and CARD domain containing | -0.052618906 | -0.043687884 | No |
| row_309 | LTBP3 | latent transforming growth factor beta binding protein 3 | -0.053137682 | -0.042533144 | No |
| row_310 | ACE | angiotensin I converting enzyme (peptidyl-dipeptidase A) 1 | -0.054349162 | -0.04436004 | No |
| row_311 | DLL4 | delta-like 4 (Drosophila) | -0.055353522 | -0.045716234 | No |
| row_312 | AZI1 | 5-azacytidine induced 1 | -0.055789925 | -0.044460684 | No |
| row_313 | IRF1 | interferon regulatory factor 1 | -0.055883069 | -0.042336542 | No |
| row_314 | GAS6 | growth arrest-specific 6 | -0.056586109 | -0.04256457 | No |
| row_315 | INSR | insulin receptor | -0.057503674 | -0.042973984 | No |
| row_316 | SGK3 | serum/glucocorticoid regulated kinase family, member 3 | -0.058041431 | -0.042497903 | No |
| row_317 | EGFR | epidermal growth factor receptor (erythroblastic leukemia viral (v-erb-b) oncogene homolog, avian) | -0.058478553 | -0.04135642 | No |
| row_318 | GRK5 | G protein-coupled receptor kinase 5 | -0.060376961 | -0.045765616 | No |
| row_319 | FRK | fyn-related kinase | -0.061014757 | -0.044743992 | No |
| row_320 | IFI30 | interferon, gamma-inducible protein 30 | -0.061028771 | -0.042424258 | No |
| row_321 | HIPK2 | homeodomain interacting protein kinase 2 | -0.061656218 | -0.04202704 | No |
| row_322 | HNF4A | hepatocyte nuclear factor 4, alpha | -0.062820427 | -0.0420181 | No |
| row_323 | HMOX1 | heme oxygenase (decycling) 1 | -0.064434484 | -0.043894175 | No |
| row_324 | null | null | -0.065371171 | -0.042274438 | No |
| row_325 | SLC9A3R1 | solute carrier family 9 (sodium/hydrogen exchanger), member 3 regulator 1 | -0.065373428 | -0.03978956 | No |
| row_326 | RUNX2 | runt-related transcription factor 2 | -0.068964988 | -0.04192595 | No |
| row_327 | RAP1GAP | RAP1 GTPase activating protein | -0.072505392 | -0.045009088 | No |
| row_328 | DLL1 | delta-like 1 (Drosophila) | -0.074289754 | -0.045429245 | No |
| row_329 | JAK3 | Janus kinase 3 (a protein tyrosine kinase, leukocyte) | -0.074945763 | -0.04344557 | No |
| row_330 | MARVELD3 | MARVEL domain containing 3 | -0.075338207 | -0.04144698 | No |
| row_331 | PGF | placental growth factor, vascular endothelial growth factor-related protein | -0.075753942 | -0.038567536 | No |
| row_332 | TGFBR3 | transforming growth factor, beta receptor III (betaglycan, 300kDa) | -0.076535545 | -0.036739696 | No |
| row_333 | PRKCZ | protein kinase C, zeta | -0.078298397 | -0.035061114 | No |
| row_334 | RBM38 | RNA binding motif protein 38 | -0.083768696 | -0.037499864 | No |
| row_335 | PLCD1 | phospholipase C, delta 1 | -0.084498733 | -0.03536934 | No |
| row_336 | MFI2 | antigen p97 (melanoma associated) identified by monoclonal antibodies 133.2 and 96.5 | -0.089282416 | -0.036733456 | No |
| row_337 | PDGFA | platelet-derived growth factor alpha polypeptide | -0.089625381 | -0.03397554 | No |
| row_338 | null | null | -0.090217195 | -0.030762605 | No |
| row_339 | BCL11B | B-cell CLL/lymphoma 11B (zinc finger protein) | -0.091834724 | -0.028353238 | No |
| row_340 | HPSE | heparanase | -0.092234984 | -0.02549613 | No |
| row_341 | EDN3 | endothelin 3 | -0.095088981 | -0.031397324 | No |
| row_342 | CXCR3 | chemokine (C-X-C motif) receptor 3 | -0.095142365 | -0.027780915 | No |
| row_343 | SCIN | scinderin | -0.09518268 | -0.0245955 | No |
| row_344 | null | null | -0.095956944 | -0.023975812 | No |
| row_345 | UTS2R | urotensin 2 receptor | -0.098464347 | -0.029099917 | No |
| row_346 | CHRNA7 | cholinergic receptor, nicotinic, alpha 7 | -0.098634958 | -0.025567017 | No |
| row_347 | OSR2 | odd-skipped related 2 (Drosophila) | -0.100635223 | -0.026067084 | No |
| row_348 | C1ORF177 | chromosome 1 open reading frame 177 | -0.10080906 | -0.022235282 | No |
| row_349 | PTCHD2 | patched domain containing 2 | -0.101456821 | -0.020325225 | No |
| row_350 | FAM59A | family with sequence similarity 59, member A | -0.103110887 | -0.02224503 | No |
| row_351 | CDKN2B | cyclin-dependent kinase inhibitor 2B (p15, inhibits CDK4) | -0.103699319 | -0.019600946 | No |
| row_352 | GCNT2 | glucosaminyl (N-acetyl) transferase 2, I-branching enzyme (I blood group) | -0.106505446 | -0.024851931 | No |
| row_353 | REG3G | regenerating islet-derived 3 gamma | -0.106561698 | -0.021017732 | No |
| row_354 | NFATC2 | nuclear factor of activated T-cells, cytoplasmic, calcineurin-dependent 2 | -0.107479692 | -0.018446218 | No |
| row_355 | C18ORF26 | chromosome 18 open reading frame 26 | -0.108176306 | -0.016497014 | No |
| row_356 | ENPP7 | ectonucleotide pyrophosphatase/phosphodiesterase 7 | -0.110041469 | -0.017504595 | No |
| row_357 | CTSL2 | cathepsin L2 | -0.113057792 | -0.021208944 | No |
| row_358 | HMX2 | homeobox (H6 family) 2 | -0.114084877 | -0.019251416 | No |
| row_359 | GPR44 | G protein-coupled receptor 44 | -0.115407534 | -0.01940624 | No |
| row_360 | RARRES1 | retinoic acid receptor responder (tazarotene induced) 1 | -0.11584805 | -0.015435327 | No |
| row_361 | LOC389493 | - | -0.117670603 | -0.01247645 | No |
| row_362 | EMX1 | empty spiracles homolog 1 (Drosophila) | -0.1189854 | -0.010765176 | No |
| row_363 | RLTPR | - | -0.12041267 | -0.009215914 | No |
| row_364 | ESR2 | estrogen receptor 2 (ER beta) | -0.122296318 | -0.007595053 | No |
| row_365 | DRD2 | dopamine receptor D2 | -0.126832366 | -0.011640875 | No |
| row_366 | HIST1H2AE | histone cluster 1, H2ae | -0.134544685 | -0.016691126 | No |
| row_367 | FGF3 | fibroblast growth factor 3 (murine mammary tumor virus integration site (v-int-2) oncogene homolog) | -0.13566421 | -0.012615778 | No |
| row_368 | TNFRSF18 | tumor necrosis factor receptor superfamily, member 18 | -0.136355102 | -0.008514169 | No |
| row_369 | ALOX15B | arachidonate 15-lipoxygenase, type B | -0.144855186 | -0.009928568 | No |
| row_370 | TP73 | tumor protein p73 | -0.145421088 | -0.004833567 | No |
| row_371 | GHRHR | growth hormone releasing hormone receptor | -0.153649464 | -0.004399851 | No |
| row_372 | TERT | telomerase reverse transcriptase | -0.155853957 | 2.27E-04 | No |
| row_373 | SLC6A4 | solute carrier family 6 (neurotransmitter transporter, serotonin), member 4 | -0.165872231 | -3.89E-04 | No |
| row_374 | PLAG1 | pleiomorphic adenoma gene 1 | -0.168125585 | 0.004920345 | No |
| row_375 | FGF9 | fibroblast growth factor 9 (glia-activating factor) | -0.183478847 | 0.006920416 | No |
